# Potential of Mean Force between Bare or Grafted Silica/Polystyrene Surfaces from Self-Consistent Field Theory

**DOI:** 10.3390/polym13081197

**Published:** 2021-04-07

**Authors:** Aristotelis P. Sgouros, Constantinos J. Revelas, Apostolos T. Lakkas, Doros N. Theodorou

**Affiliations:** School of Chemical Engineering, National Technical University of Athens (NTUA), GR-15780 Athens, Greece; cjrevelas@gmail.com (C.J.R.); tolis1981@gmail.com (A.T.L.)

**Keywords:** SCF, PMF, brushes, polymer, agglomeration

## Abstract

We investigate single and opposing silica plates, either bare of grafted, in contact with vacuum or melt phases, using self-consistent field theory. Solid–polymer and solid–solid nonbonded interactions are described by means of a Hamaker potential, in conjunction with a ramp potential. The cohesive nonbonded interactions are described by the Sanchez-Lacombe or the Helfand free energy densities. We first build our thermodynamic reference by examining single surfaces, either bare or grafted, under various wetting conditions in terms of the corresponding contact angles, the macroscopic wetting functions (i.e., the work of cohesion, adhesion, spreading and immersion), the interfacial free energies and brush thickness. Subsequently, we derive the potential of mean force (PMF) of two approaching bare plates with melt between them, each time varying the wetting conditions. We then determine the PMF between two grafted silica plates separated by a molten polystyrene film. Allowing the grafting density and the molecular weight of grafted chains to vary between the two plates, we test how asymmetries existing in a real system could affect steric stabilization induced by the grafted chains. Additionally, we derive the PMF between two grafted surfaces in vacuum and determine how the equilibrium distance between the two grafted plates is influenced by their grafting density and the molecular weight of grafted chains. Finally, we provide design rules for the steric stabilization of opposing grafted surfaces (or fine nanoparticles) by taking account of the grafting density, the chain length of the grafted and matrix chains, and the asymmetry among the opposing surfaces.

## 1. Introduction

Grafting polymer chains on solid surfaces is a standard procedure for the steric stabilization of nanocomposite systems [1,2,3]. Various methods for the experimental synthesis of such systems are reported in the literature [4,5,6]. Understanding the behavior of grafted polymer brushes requires a thorough investigation of the thermodynamics of the system under different conditions. In the case of two or more nanoparticles embedded inside a polymer melt, the challenge is to keep them separated by overcoming their tendency to form aggregates. When nanoparticles are bare, the attractive Van der Waals forces [7] drive them to come closer to each other. One of the possible ways to get around this behavior is to graft polymer chains on the surface of the nanoparticles. Achieving a proper dispersion of nanoparticles inside the polymer melt is associated with a considerable enhancement of its properties [8,9,10,11,12].

Major computational research has been conducted on systems comprising a single grafted nanoparticle embedded in a solvent or a homopolymer matrix, using theoretical formulations [13,14] or atomistic simulations [15,16,17,18]. Moreover, considerable work has addressed the behavior of grafted and matrix chains in systems comprising multiple grafted solid surfaces [19,20,21,22,23,24,25]. Munao et al. [19] demonstrated the effect of a third nanoparticle, when inserted in a system of two interacting grafted silica nanoparticles, while Martin et al. [23] investigated the effect of polydispersity of grafted chains on the structural properties of the nanocomposite system.

In this work, we undertake a self-consistent field theory [26,27,28] (SCFT)-based analysis to examine the structural properties and thermodynamics of polystyrene (PS) melt (matrix) confined between two silica plates, either grafted or bare, as well as of the same plates in the absence of melt. In fact, when no matrix chains are present between the two grafted surfaces, or they are present but their molecular weight is significantly larger than that of the grafted chains, the latter behave as if they were in contact with a very bad solvent. Following Flory’s theory [29], when matrix chains are present and their molecular weight is similar to the one of grafted chains, then the system is analogous to one wherein the grafted chains are embedded inside a theta solvent. On the contrary, when matrix chains are much shorter than the grafted chains, the latter are starting to swell towards the matrix as if they were in contact with a good solvent.

Specifically, we derive the potential of mean force between the plates by varying the distance between them. Studying the thermodynamics in such a planar geometry is quite important in the field of biomembranes and other biological applications [30,31]. One could consider this planar geometry study as the equivalent of investigating the potential of mean force between spherical (fine) particles, whose radius is large enough, in comparison to chain dimensions, for their curvature to be negligible. Materials consisting entirely of matrix-free grafted plates or hairy nanoparticles (also referred to as “particle solids”) exhibit interesting mechanical and optical properties, while they behave as tough glasses when assembling in specific configurations [32,33,34,35,36]. Barnett and Kumar [37,38] have published several works, where they report the use of such materials in the design of membranes for separation processes. In their recent work, Biltchak et al. [39] studied the effect of the addition of matrix chains to a neat grafted nanoparticle-based membrane on its selectivity in separations of gases of different molecular size. Mydia et al. [40] developed a two-layer theoretical model to describe the configurations of the grafted chains in the vicinity of the grafted nanoparticles and at intermediate distances between them and they compared their model with atomistic molecular dynamics simulations.

The problem of polymer grafted chains in planar geometries has been addressed in the past by several works [41,42]. By removing the incompressibility assumption and imposing Dirichlet boundary conditions at the solid surfaces, we make a step forward towards the investigation of systems with realistic interfacial free energies. Furthermore, we explicitly describe the solid–polymer and solid–solid interactions via the Hamaker potential, and we explore its influence on the potential of mean force. Without these considerations, we would not be able to apply our methodology in systems comprising exclusively grafted chains, i.e., systems in the absence of polymer matrix chains, since the incompressibility condition requires that the total segment density profile be uniform across the entire domain of interest [43]. Furthermore, such approaches—when applied to three dimensions—allow for the investigation of systems of complex geometry, where the use of Fourier based methods is not recommended, since no symmetry appears and no periodic boundary conditions can be implemented.

In the majority of computational works, it is assumed that the plates have the same grafting density and molecular weight as the grafted chains [41,42]. In reality, it is rather hard for experimentalists to prepare such a perfectly symmetric system. Herein, we increase the degrees of freedom of the system by allowing the grafted chains on each plate to have different molecular weights, whereas each plate may have its own grafting density as well. The goal is to reveal the influence of these kinds of asymmetries on the potential of mean force between the two grafted plates when varying the distance between them, and to propose a scaling law that accounts for all of these design aspects.

As a first step, we build our thermodynamic reference system by deriving the free energy of single bare or grafted plates, either isolated or in contact with polystyrene melt. Next, we demonstrate the potential of mean force of a system which contains matrix chains exclusively. The melt is assumed to be at equilibrium with a bulk melt phase at all times and it is gradually squeezed by reducing the distance between the two silica surfaces. Finally, we demonstrate results concerning the structure and potential of mean force in a system of two grafted silica plates over a large parameter space, involving the molecular weight of matrix chains, the molecular weight of grafted chains on each plate, and the grafting density of each plate. Understanding the behavior of matrix and grafted chains in this planar geometry is a stepping-stone towards the corresponding spherical case, i.e., two or more polystyrene-grafted silica nanoparticles embedded in a polystyrene matrix.

In the context of our SCFT calculations, we employ the Sanchez-Lacombe (SL) equation of state in conjunction with square gradient theory (SGT), which has been shown to reproduce correctly the density profiles and surface tension of a number of different industrially important thermoplastic polymers, when compared with atomistic molecular dynamics simulations or experiment [44]. In this work, we introduce an additional potential term which allows for a refinement of the polymer–solid interactions; by adjusting the strength of these interactions, we examine interphases with low (LW), high (HW) and perfect (PW) wetting. For comparison purposes, we evaluate several of our findings with Helfand’s (HFD) free energy density [45] as well, using a constant compressibility [15]; this free energy density is most commonly used in SCFT studies whenever the incompressibility condition is relaxed. It is noted at this point, that in cases where matrix chains are not present in the system, only the SL EoS is applicable, since HFD fails to describe correctly the density profiles of grafted chains in contact with vacuum and the corresponding free energy of the system.

It is mentioned here that, since the employed model is one-dimensional, a smearing of the grafting points parallel to the solid surfaces is required. As discussed in our previous work [46], this smearing may become an inaccurate approximation when addressing grafted surfaces with low *σ*_g_*R*_g_^2^ values (*σ*_g_ being the surface grafting density and *R*_g_^2^ the mean squared radius of gyration of grafted chains) and small nanoparticles (large curvatures). Nonetheless, the effect of smearing is weaker in the present work, since it deals with planar surfaces (zero curvature) where it is less probable for the grafted chains to adopt a mushroom-like configuration.

The manuscript is structured as follows. Section 2 discusses the systems under study, presents the formulation for grafted chains in the presence and in the absence of a melt phase, whereas Section 3 provides details regarding the calculations. The first part of the Results section (Section 4.1) discusses single surfaces, either bare (polymer/vacuum and polymer/solid interphases), or grafted and in the presence or absence of melt chains. The structural and thermodynamic properties derived from these systems are used as reference quantities for the analysis of two opposing surfaces. Subsequently, we present results regarding the potential of mean force (PMF) of approaching silica surfaces, either bare (Section 4.2), or grafted, in the presence (Section 4.3) or absence (Section 4.4) of melt chains.

## 2. Model and Theoretical Formulation

### 2.1. Model Systems

**Figure 1:**
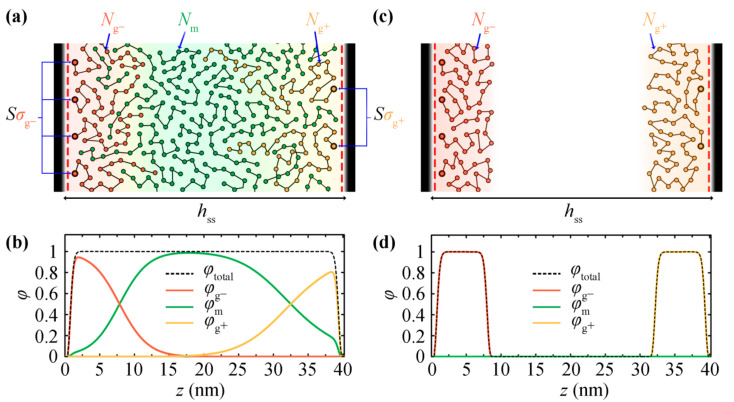
(**a**) Bead-spring representation of two opposing grafted silica walls embedded in a melt (GMG system) comprising matrix chains of length $N_{m}$. The silica wall on the left (right) is grafted with $\sigma_{g^{-}}S_{\mathrm{solid}}$ ($\sigma_{g^{+}}S_{\mathrm{solid}}$) grafted chains of length $N_{g^{-}}$ ($N_{g^{+}}$). (**c**) The same system as (**a**), but in absence of matrix chains (GVG system). Additionally, the corresponding reduced density profiles from the opposing grafted silica walls in the (**b**) presence and (**d**) absence of the matrix chains are shown.

Figure 1 illustrates a mesoscopic bead-spring representation of the systems under study. Two opposing silica plates at distance *h*_ss_ are grafted with polystyrene chains, while matrix chains may or may not exist in the space between the grafted plates. Hereafter, the matrix chains are denoted by m, the chains grafted on the left silica plate are denoted by g^–^, and the chains grafted on the right silica plate are denoted by g^+^. When polymer melt exists between the two plates, it comprises a total number of *n*_m_ monodispersed chains of length equal to *N*_m_ monomers/segments. The melt, when present, is at equilibrium with a bulk polymer phase of chain length *N*_m_ of temperature *T* and pressure *P*. The left/right surfaces are grafted with $n_{g^{-}}$/$n_{g^{+}}$ chains of length $N_{g^{-}}$/$N_{g^{+}}$ segments, whereas the corresponding grafting density equals $\sigma_{g^{\mp }}=n_{g^{\mp }}/S_{\mathrm{solid}}$ with $S_{\mathrm{solid}}$ denoting the area of each surface. The grafting density and molecular weight of grafted chains are allowed to vary between the two plates. It is obvious from the density profiles in Figure 1b,d—corresponding to systems with Figure 1a and without Figure 1c matrix chains—that, in the former case, the extension of each brush towards the opposing plate is favored.

In the rest of this manuscript, the situation at the boundaries will be referred to either as V (vacuum), S (bare solid surface) or G (grafted solid surface), and the intermediate region between the plates will be referred to as V (vacuum) or M (melt). For example, systems comprising vacuum/melt, bare solid/melt, grafted solid/melt and grafted solid/vacuum phases will be referred to as VM, SM, GM and GV, respectively. In the same spirit, more complex systems: solid/melt/solid, grafted/melt/grafted (Figure 1a) and grafted/vacuum/grafted (Figure 1b) will be referred to as SMS, GMG and GVG, respectively.

### 2.2. Self-Consistent Field Convergence Scheme

The polymer chains are described with the Gaussian thread model. Consequently, their propagation inside a three-dimensional space is described by the Edwards diffusion equation [47] subject to the field ${w^{\prime }}_{\mathrm{ifc}}$:

(1) $$\frac{\partial }{\partial N}q_{c}(r,N)=\frac{R_{G,c}{}^{2}}{N_{c}}\nabla_{r}^{2}q_{c}(r,N)-\beta {w^{\prime }}_{\mathrm{ifc}}(r)q_{c}(r,N)\;(c{\;=\;m,\;g}^{-}{,\;g}^{+})$$

where $q_{c}$ is the restricted partition function, $R_{G,c}$ is the radius of gyration of chains, $N_{c}$ is the chain length measured in monomer units, and *c* denotes the kind of the chains (m: matrix, g^–^/g^+^: chains grafted to the left/right side). Unless otherwise stated, the initial configuration of the field is set equal to zero across the domain. For more details concerning the iterative solution of Equation (1) in the presence of the field, the reader is referred to Section 2.1.1 of Reference [46]. In the Section 2.1.2 of the same work [46], we explain how Equation (1) reduces to its one-dimensional analogue, and in its supporting information we elaborate on the numerical solution of Equation (1) via a finite difference scheme and on the stability criteria for the proper equilibration of the field ${w^{\prime }}_{\mathrm{ifc}}$.

Dirichlet boundary conditions are imposed on the edges of the domain when they correspond to solid or gas phases and Neumann (with zero flux) when they correspond to bulk polymer phases (in cases of single interfaces). The initial conditions of matrix chains are given by $q_{m}(r,0)=1$, while for grafted chains the following Equation (2) holds in an one-dimensional domain.

(2) $$q_{g}(h_{g},0)=\frac{S_{\mathrm{solid}}}{S_{h_{g}}}\frac{\sigma_{g}N_{g}}{\rho_{\mathrm{seg},\mathrm{bulk}}}\frac{\delta \left(h-h_{g}\right)}{q_{m}\left(h_{g},N_{g}\right)}$$

with $S_{h_{g}}$ being the surface area of the plane over which the smearing of grafting points takes place (equal to $S_{\mathrm{solid}}$ in planar geometries), and $q_{m}(h_{g},N_{g})$ is the chain propagator for matrix chains with length *N*_g_ at distance *h*_g_ from a solid plate. The delta function $\delta \left(h-h_{g}\right)$ is analytically evaluated as the inverse of the width Δ*h* of the interval assigned to the points of the mesh which are closest to the two solid surfaces and is zero everywhere else.

The reduced density, $\varphi_{c}=\rho_{c}/\rho_{\mathrm{seg},\mathrm{bulk}}$, can be calculated from the corresponding restricted partition function using the convolution integral in the following Equation (3).

(3) $$\varphi_{c}(r)=\frac{1}{N_{c}}\int_{0}^{N_{c}}dN\;q_{c}(r,N)\;q_{m}(r,N_{c}-N)\;(c={m,\;g}^{-}{,g}^{+})$$

with *ρ*_seg,bulk_ = *ρ*_mass,bulk_ *N*_A_/*m*_monomer_ being the segmental number density in the bulk. With *φ_c_* known for each chain type, the field is estimated based on the free energy density, f[ρ,∇ρ], from a suitable EoS:

(4) $${w^{\prime }}_{\mathrm{ifc}}(r)=w^{\prime }(r)-{w^{\prime }}_{\mathrm{bulk}}={\left.\frac{\partial f[\rho ,\nabla \rho ]}{\partial \rho }\right|}_{\rho =\rho (r)}-{\left.\frac{\partial f[\rho ,\nabla \rho ]}{\partial \rho }\right|}_{\rho =\rho_{\mathrm{seg},\mathrm{bulk}}}-\nabla \cdot \frac{\partial f[\rho ,\nabla \rho ]}{\partial \nabla \rho }+u_{s}(r)$$

For more details about the corresponding EoS, the reader is referred to Section 2.4.1, Section 2.4.2 and Section 2.4.3.

### 2.3. Thermodynamic Description

#### 2.3.1. Systems with Melt: Grand Canonical Ensemble

Following the formulation developed in our previous work [46], when melt/matrix chains exist between the two plates, then we describe the equilibrium of the system in the context of the grand canonical ensemble. Hence, the thermodynamics of the polymer-grafted planar surfaces in contact with a matrix phase is described by a grand potential of the form:

(5) $$\Delta \Omega =\Omega -\Omega_{\mathrm{bulk}}-A_{g,\mathrm{bulk}}=\Delta \Omega_{\mathrm{coh}}+\Delta \Omega_{\mathrm{field}}+\Delta \Omega_{m}+\Delta A_{g}+U_{s}+U_{\mathrm{ss}}$$

Note that the grand potential has been defined with respect to a reference system at the same temperature, which comprises the two solids at infinite distance from each other so that they do not interact, a bulk phase of matrix chains of length $N_{m}$ with chemical potential $\mu_{m}N_{m}$, occupying the same polymer-accessible volume as the interfacial system, and a set of $n_{g^{-}}$ and $n_{g^{+}}$ unperturbed end-pinned grafted chains of length $N_{g^{-}}$ and $N_{g^{+}}$, respectively, exposed to bulk polymer. The segment density of the bulk polymer at temperature *T* and chemical potential $\mu_{m}N_{m}$ is $\rho_{\mathrm{seg},\mathrm{bulk}}$.$\Delta \Omega_{\mathrm{coh}}$ in Equation (5) describes the nonbonded cohesive interactions among the polymer segments as governed by the free energy density of the equation of state:

(6) $$\Delta \Omega_{\mathrm{coh}}=\int_{R}dr\left\{f\left[\rho (r),\nabla \rho (r)\right]-f\left[\rho_{\mathrm{seg},\mathrm{bulk}},0\right]\right\}$$

$\Delta \Omega_{\mathrm{field}}$ describes the interaction between the chemical potential field and the density field [26],

(7) $$\Delta \Omega_{\mathrm{field}}=-\int_{R}dr\left\{\rho (r)w^{\prime }(r)-\rho_{\mathrm{seg},\mathrm{bulk}}{w^{\prime }}_{\mathrm{bulk}}(r)\right\}$$

$\Delta \Omega_{m}$ encompasses the free energy associated with the translational and conformational entropy (with respect to the entropy of a bulk melt) of matrix chains and it is given by the following Equation (8).

(8) $$\Delta \Omega_{m}=-\frac{\rho_{\mathrm{seg},\mathrm{bulk}}V}{\beta N_{m}}\left(Q_{m}\left[{w^{\prime }}_{\mathrm{ifc}}\right]-1\right)$$

$\Delta A_{g}$ is associated with the conformational entropy of $n_{g^{-}}$ and $n_{g^{+}}$ grafted chains subject to the field ${w^{\prime }}_{\mathrm{ifc}}$, and it is given by Equation (9).

(9) $$\begin{array}{ll} \Delta A_{g}= & \Delta A_{g^{-}}+\Delta A_{g^{+}}\\ & =-\frac{1}{\beta }\sum_{i_{g^{-}}=1}^{n_{g^{-}}}\ln Q_{g}\left[r_{g,i_{g^{-}}};{w^{\prime }}_{\mathrm{ifc}}\right]-\frac{1}{\beta }\sum_{i_{g^{+}}=1}^{n_{g^{+}}}\ln Q_{g}\left[r_{g,i_{g^{+}}};{w^{\prime }}_{\mathrm{ifc}}\right]\\ & -\frac{1}{\beta }\sum_{i_{g}=1}^{n_{g^{-}}+n_{g^{+}}}\ln\frac{r_{\mathrm{ref},q=0}^{}}{r_{g,i_{g},q=0}^{}} \end{array}$$

Note that the last term in the right-hand side of Equation (9) is added in order to normalize the free energy term, $\Delta A_{g}$, with respect to the distance of the grafting point from the solid surface and therefore render it independent of the spatial discretization of the system [46].

$U_{s}$ is the interaction energy between the polymer chain segments and the left and right solid surfaces,

(10) $$U_{s}=U_{s^{-}}+U_{s^{+}}=\int_{R}dr\left\{\rho (r)u_{s^{-}}^{}(r)\right\}+\int_{R}dr\left\{\rho (r)u_{s^{+}}^{}(r)\right\}$$

$U_{\mathrm{ss}}$ describes the direct interactions between the opposing semiinfinite solid surfaces. Expressions for *u*_s_(**r**) and *U*_ss_ are given in Section 2.5.1 and Section 2.5.2 below.

For the full SCFT formulation, the reader is referred to Reference [46] and its supporting information.

#### 2.3.2. Systems without Melt: Canonical Ensemble

In order to address the system where no matrix chains exist, we need to modify the above formulation and describe the thermodynamics of the system by means of the canonical ensemble. In more detail, the thermodynamics of a melt-free system with polymer chains grafted to a surface is described by the following expression for the Helmholtz free energy,

(11) $$\Delta A=A-A_{\mathrm{bulk}}=\Delta A_{\mathrm{coh}}+\Delta A_{\mathrm{field}}+\Delta A_{g}+U_{\mathrm{solid}}+U_{\mathrm{ss}}$$

with *A*_bulk_ being the Helmholtz energy of a reference system at the same temperature, consisting of the two solids at infinite distance from each other and a set of $n_{g^{-}}$ and $n_{g^{+}}$ unperturbed end-pinned grafted chains exposed to bulk melt with length $N_{g^{-}}$ and $N_{g^{+}}$, respectively [46]. The first term on the right-hand side of Equation (11) describes the cohesive interactions among the polymer segments as governed by the free energy density of the equation of state,

(12) $$\Delta A_{\mathrm{coh}}=\int_{R}dr\left\{f\left[\rho (r),\nabla \rho (r)\right]\right\}$$

the second term describes the interaction between the chemical potential field and the density field [26]:

(13) $$\Delta A_{\mathrm{field}}=-\int_{R}dr\left\{\rho (r)w^{\prime }(r)\right\}$$

while the rest of the terms remain the same as those in Equation (5).

Since the article addresses planar geometries, it would be meaningful to report the free energies per unit area. Hereafter, the free energy terms in Equations (5)–(13) will be referred to as:

(14) $$\gamma_{\alpha }^{\mathrm{sys}}=E_{\alpha }^{\mathrm{sys}}/S_{\mathrm{solid}}$$

where *E* is Ω, *A*, or *U*, “α” denotes the kind of contribution (coh, field, m, g^−/+^ and s^−/+^) whereas omitting “α” corresponds to the total free energy. The “sys” symbol denotes the kind of system (see Section 2.1) and hence implicitly the statistical ensemble; i.e., a system with/without melt is described in the grand canonical/canonical ensemble. A few examples: $\gamma_{}^{\mathrm{VM}}$, the total free energy of a melt in contact with vacuum (surface tension); $\gamma_{}^{\mathrm{SM}}$, the total free energy of a melt in contact with a silica plate (minus adhesion tension); $\gamma_{\mathrm{coh}}^{\mathrm{GVG}}=\Delta A_{\mathrm{coh}}/S_{\mathrm{solid}}$, the contribution from cohesive interactions in the polymer to the Helmholtz energy of a system consisting of two grafted silica surfaces with vacuum in between them; $\gamma_{\mathrm{coh}}^{\mathrm{GMG}}=\Delta \Omega_{\mathrm{coh}}/S_{\mathrm{solid}}$, the cohesive term for a melt capped between two grafted silica surfaces; etc.

### 2.4. Nonbonded Free Energy Density

The following subsections illustrate the contributions of the square gradient theory and the free energy density functionals to the cohesive (Equations (6) and (12)) and field (Equations (7) and (13)) terms. These equations represent the nonbonded interactions between polymer segments in the context of the field theoretical formulation applied herein.

#### 2.4.1. Square Gradient Theory

Given the steepness of the field and the segment density profiles, we expand the free energy density functional governing the nonbonded interactions by considering it a functional of the density gradient too, as shown in the following Equation (15) [48,49]. The contribution of the gradient term to the free energy of the system is weighted by the influence parameter, *κ*. The value of this parameter is reported in Table 1. It has been obtained after a thorough comparison of SCFT with atomistic molecular dynamics simulations conducted by the authors in the past for a wide range of polymer melts [44].

(15) $$f\left[\rho (r),\nabla \rho (r)\right]=f_{\mathrm{EoS}}\left(\rho (r)\right)+\frac{1}{2}\kappa {\left(\nabla \rho (r)\right)}^{2}$$

(16) $${w^{\prime }}_{\mathrm{ifc}}(r)={\left.\frac{\partial f_{\mathrm{EoS}}(\rho )}{\partial \rho }\right|}_{\rho =\rho (r)}-{\left.\frac{\partial f_{\mathrm{EoS}}(\rho )}{\partial \rho }\right|}_{\rho =\rho_{\mathrm{seg},\mathrm{bulk}}}-\kappa {\left.\nabla^{2}\rho \right|}_{\rho =\rho (r)}+U_{s}(r)$$

#### 2.4.2. Sanchez-Lacombe

The Sanchez-Lacombe (SL) equation of state [50] and the corresponding free energy density are given by Equations (17) and (18), respectively. The symbols $\tilde{\rho }=\rho /\rho^{*}$, $\tilde{P}=P/P^{*}$, and $\tilde{T}=T/T^{*}$ represent the reduced density, pressure and temperature, respectively, where ρ^*^, P^*^, and T^*^ correspond to the characteristic SL density, pressure and temperature. For polystyrene, the values of those characteristic parameters are reported in Table 1.

(17) $${\tilde{\rho }}^{2}+\tilde{P}+\tilde{T}\left[\ln(1-\tilde{\rho })+\left(1-\frac{1}{r_{\mathrm{SL}}}\right)\tilde{\rho }\right]=0$$

(18) $$f_{\mathrm{EoS}}^{\mathrm{SL}}\left(\rho \left(r\right)\right)=P^{*}\left[\tilde{T}\tilde{\rho }-{\tilde{\rho }}^{2}+\tilde{T}(1-\tilde{\rho })\ln(1-\tilde{\rho })\right]$$

Using Equation (17), we can derive the mass density across the bulk fluid phase, *ρ*_mass,bulk_, for each chain length from the vapor-liquid equilibrium of a SL fluid; more details in the Supplemental Material Section S1 in Reference [44].

#### 2.4.3. Helfand Free Energy Density

A simpler EoS which can be used in the context of a compressible model for the description of the nonbonded polymer interactions is the Helfand (HFD) equation appearing in the following Equation (19) [45].

(19) $$f_{\mathrm{EoS}}^{\mathrm{HFD}}\left(\rho \left(r\right)\right)=\frac{1}{2\kappa_{T}}{\left(\frac{\rho \left(r\right)}{\rho_{\mathrm{seg},\mathrm{bulk}}}-1\right)}^{2}$$

where *κ_T_* is the isothermal compressibility of the polymer and *ρ*_seg,bulk_ is the polymer segment density in the bulk melt region. For a detailed comparison between the SL and HFD equations of state, the reader is referred to previous works of the authors [44].

### 2.5. Interactions with the Solid Surfaces

#### 2.5.1. Polymer–Solid Interactions

In the context of Hamaker theory [7], the attractive and repulsive interactions between a sphere of radius *a*_M_ and a semi-infinite solid surface are given by Equations (20) and (21).

(20) $$u_{A}^{\mathrm{SM}}=-\frac{A_{\mathrm{SM}}}{6}\left(\frac{1}{r^{\prime }}+\frac{1}{2+r^{\prime }}+\ln\left(\frac{r^{\prime }}{2+r^{\prime }}\right)\right)$$

(21) $$u_{R}^{\mathrm{SM}}=\frac{A_{\mathrm{SM}}}{7560}\frac{\sigma_{\mathrm{SM}}^{6}}{a_{M}{}^{6}}\left(\frac{8+r^{\prime }}{{\left(2+r^{\prime }\right)}^{7}}+\frac{6-r^{\prime }}{{r^{\prime }}^{7}}\right)$$

with $A_{\mathrm{SM}}=\sqrt{A_{S}A_{M}}$, $r^{\prime }=d_{\mathrm{SM}}/a_{M}$, $d_{\mathrm{SM}}=h–a_{M}$ being the distance between the surface of the sphere and the solid surface, *h* being the distance of the center of the sphere from the surface, and $\sigma_{\mathrm{SM}}=0.5\left(\sigma_{S}+\sigma_{M}\right)$ with *σ*_S_ and *σ*_M_ being the collision diameter of Lennard-Jones particles constituting the solid and melt, respectively. Even though the Hamaker potential allows for a proper description of the long-range interactions, it cannot by itself restore the proper interfacial thermodynamics of the system, due to spurious interactions with the self-consistent field, which can result in incorrect adhesion tension and density profiles. This issue prevails regardless of the EoS and whether SGT is enabled, as we demonstrate later on. Therefore, to circumvent the aforementioned limitations, we introduced an additional potential term which is given by Equation (22).

(22) $$u_{\mathrm{ramp}}^{}=v_{\mathrm{ramp}}\max\left(\frac{\sigma_{\mathrm{ramp}}-h}{\sigma_{\mathrm{ramp}}},0\right)$$

This term has a local character and by adjusting *v*_ramp_, it allows to tune the polymer–solid interactions in the vicinity of the interface and restore the proper interfacial properties of the system.

Putting it all together, in this work the interactions between the polymer chains and the solid surface are described by the Hamaker potential in conjunction to the ramp potential:

(23) $$u_{s}=u_{A}^{\mathrm{SM}}+u_{R}^{\mathrm{SM}}+u_{\mathrm{ramp}}^{}$$

Hence allowing to preserve the long-range interactions and at the same time the short-range structure and thermodynamics.

#### 2.5.2. Solid–Solid Interactions

In the context of Hamaker theory, the interactions between two semi-infinite solid surfaces separated by a medium are described by the following equation:

(24) $$U_{\mathrm{ss}}(h_{\mathrm{ss}})=S_{\mathrm{solid}}\frac{A_{\mathrm{SMS}}}{\pi }\left[\frac{\sigma_{S}{}^{6}}{360h_{\mathrm{ss}}{}^{8}}-\frac{1}{12h_{\mathrm{ss}}{}^{2}}\right]$$

The Hamaker constant for these interactions is usually approximated as $A_{\mathrm{SMS}}\approx A_{S}+A_{M}-2\sqrt{A_{S}A_{M}}$ [51]. This approximation is, however, inappropriate for describing the surface-surface interactions in our case, since the polymer/solid interactions are computed explicitly for each surface and it would lead to double counting of these interactions. In other words, the aforementioned approximation accounts—apart from the solid–solid interactions—for the polymer/solid interactions in an effective manner [51]. This can be understood more clearly by the following example:

In the trivial case where the solid and medium phases are exactly the same (in terms of structure and chemical constitution, *A*_S_ = *A*_M_), the total free energy balance (sum of the polymer–solid and solid–solid interactions) assumes a constant value that is independent of *h*_ss_. In that case, using the above approximation, *A*_SMS_ = 0, and therefore, $U_{\mathrm{ss}}=0$. However, the medium–solid interactions arising from the integration of Equation (10) vary with *h*_ss_, indicating that the free energy balance is not satisfied.

Since in our implementation the polymer–solid interactions are described explicitly, the proper treatment for *A*_SMS_ is to set it *A*_SMS_ *= A*_S_. This way, all kinds of interactions (polymer–solid and solid–solid) are calculated in an explicit manner and there are no double counting issues.

## 3. Calculation Details

The calculations have been performed with our in-house *RuSseL* code for a one-dimensional formulation. The same code has been extended to address arbitrary 3D geometries as well [52]. The values of the parameters that we used in our SCFT calculations are presented in the following Table 1. The temperature, *T*, of the system was always equal to 500 K and when matrix chains exist in the system, the calculations were performed under the grand canonical ensemble, which we find most suitable in such interfacial solid–polymer systems. On the other hand, in absence of matrix chains, we employ the canonical ensemble to describe the equilibrium properties of the system.

The position of the grafting point is set equal to the coordinate of the first discretization node of the one-dimensional spatial mesh. Ideally, the grafting point should be attached exactly on the solid surface, but numerical issues would occur, since Dirichlet boundary conditions are imposed on the solid; hence the propagator of matrix chains appearing in the denominator of Equation (2) becomes zero on the solid surface [41,58]. In our work, we use an alternative approach to address the boundary conditions at the interfaces, rather than applying reflecting boundary conditions [41]. Mathematically preventing all chains from touching the solid surfaces via Dirichlet boundary conditions, while preserving the severity of delta function initial conditions, may seem to be an overkill in such one-dimensional and symmetric geometry systems, but it is necessary if complex systems of arbitrary geometry, dimensionality or chemistry are to be addressed in the future.

The intensity of PS–SiO_2_ interactions in the context of the Hamaker potential are dictated by the corresponding $\sigma_{\mathrm{PS}}$, $A_{\mathrm{PS}}$ and $\sigma_{{\mathrm{SiO}}_{2}}$, $A_{{\mathrm{SiO}}_{2}}$ parameters, whose values are reported in Table 1. The effective radius of a PS segment was estimated as $a_{\mathrm{PS}}=\sqrt[{3}]{3/\left(4\pi \rho_{\mathrm{seg},\mathrm{bulk}}\right)}$. The range (*σ*_ramp_) of the ramp potential was set to a distance where intensity of the Hamaker potential becomes equal to –0.005 *k*_B_*T* (see critical adsorption distance in Reference [46]), and the well depth to *v*_ramp_ = 0 J, –2.481 × 10^−20^ J and –3.975 × 10^−20^ J for cases of low (LW), high (HW) and perfect (PW) wettability conditions [54], respectively. *v*_ramp_ were optimized for each *W*_A_ value via the secant optimization method. Retaining the full repulsive part of this potential would result in a very steep configuration of the self-consistent field near the solid surface. For that reason, we replace the repulsive part of the solid potential with a repulsive wall positioned at a distance equal to, *h*_HS_ ~ 0.4 nm, from the solid silica plate, where it assumes a value of *u*_s_(*h*_HS_) = 5 *k*_B_*T* (exactly). Unless otherwise stated, the evaluations will be performed in the absence of the ramp potential, whereas the solid–solid interactions are active in all cases of two opposing solid surfaces.

## 4. Results

### 4.1. Single Surfaces

The present section discusses the thermodynamics and structure (in terms of the brush dimensions) of vacuum/melt (VM), solid/melt (SM), grafted/melt (GM) and grafted/vacuum (GV) interphases. The quantities extracted from these systems will be used as reference for the potential of mean force calculations involving two approaching silica surfaces, either grafted or bare.

#### 4.1.1. Vacuum/Melt (VM) and Solid/Melt (SM) Interphases

**Figure 2:**
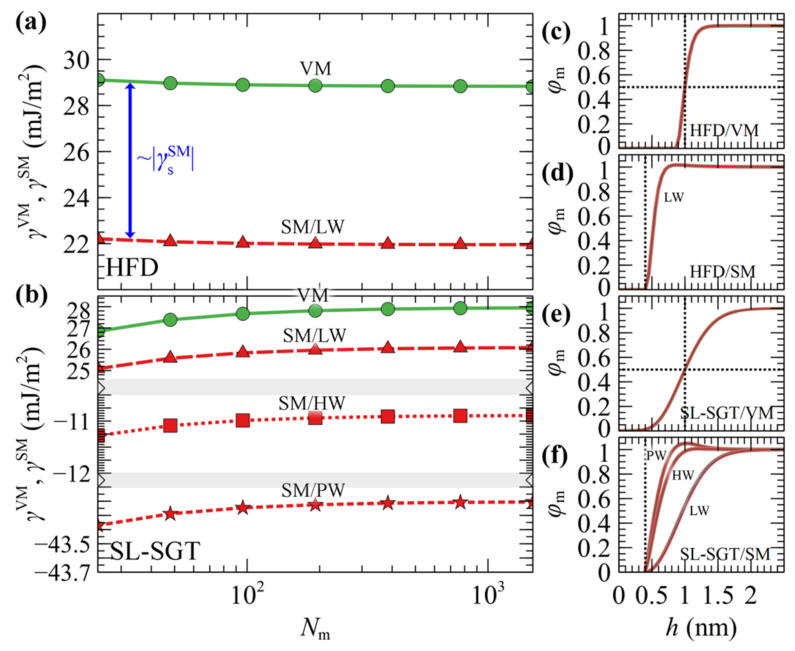
The free energy of vacuum/melt (VM, circles) and bare solid/melt (SM) with low wetting (LW, triangles), high wetting (HW, squares) and perfect wetting (PW, stars) wetting, as a function of chain length using the (**a**) HFD and (**b**) SL-SGT EoS. Bands denote scale changes along the axes. The right panels depict the corresponding reduced density profiles of VM and SM interphases with HFD (**c**,**d**) and SL-SGT (**e**,**f**) for *N*_m_ = 24, 48, 96, 192, 384, 768 and 1536. Even though the thickness of the lines increases with increasing *N*_m_, there is practical coincidence of reduced density profiles for all chain lengths; thus, the reader has to zoom considerably to notice any deviations. The dotted lines in (**c**–**f**) are guides to the eye.

Figure 2 presents the free energy of VM (surface tension) and SM (minus adhesion tension) interphases as a function of chain length. In Figure 2a, the free energies have been evaluated with HFD. In Figure 2b the SL-SGT model is considered with the original Hamaker potential corresponding to low wetting conditions (LW), and with the addition of the ramp potential that has been adjusted to reproduce the work of adhesion of PS melts in contact with treated (high wetting, HW) and untreated (perfect wetting, PW) silica [54].

In interphases with low wetting, the free energies are qualitatively similar for VM and SM; they differ by about $\gamma_{s}^{\mathrm{SM}}$ across the chain molar mass range explored herein. This is attributed to the fact that the density profiles for these cases are very similar (e.g., compare Figure 2c with Figure 2d, and Figure 2e with the “LW” curve in Figure 2f); thus, any differences in the free energies can be attributed to the polystyrene/silica Hamaker interactions.

Increasing the strength of the ramp potential enhances the polymer–solid interactions as indicated by the more negative free energies in Figure 2b, and the more pronounced peaks of the density profiles in Figure 2f.

The free energy from SL in Figure 2b appears to be an increasing function of chain length, and this behavior is anticipated, since the cohesion of the polymer increases with increasing chain length [59,60,61]. In contrast, HFD (Figure 2a) exhibits the opposite trend and this is attributed to the fact that all evaluations have been performed using a constant isothermal compressibility. In Section S1 of the Supplementary Materials we demonstrate that tuning the HFD compressibilities (with and without employing SGT) based on the predictions of SL, or even fitting them directly to the experimental surface tensions, allows to restore the proper chain length dependence in the thermodynamic behavior of the systems in terms of the interfacial free energies. Furthermore, as shown in Section S2 of the Supplementary Materials, for the longer chain lengths considered here, one must apply special care to converge the SCF efficiently; the density profiles tend to shift further towards the bulk region with increasing *N*_m_ and, as a result, the SCF is prone to entrapment in metastable states for a large number of iterations.

The thermodynamics of these films can be understood better in terms of the four macroscopic wetting functions, i.e., the work of cohesion (*W*_C_), the work of adhesion (*W*_A_), the work of spreading (*W*_S_) and the work of immersion (*W*_I_):

(25) $$W_{C}=2\gamma^{\mathrm{VM}}=2\sigma^{\mathrm{VM}}$$

(26) $$W_{A}=\gamma^{\mathrm{VM}}-\gamma^{\mathrm{SM}}=\sigma^{\mathrm{VM}}+\sigma^{\mathrm{SV}}-\sigma^{\mathrm{SM}}=\sigma^{\mathrm{VM}}\left(\cos\theta +1\right)$$

(27) $$W_{S}=-\gamma^{\mathrm{VM}}-\gamma^{\mathrm{SM}}=-\sigma^{\mathrm{VM}}+\sigma^{\mathrm{SV}}-\sigma^{\mathrm{SM}}=\sigma^{\mathrm{VM}}\left(\cos\theta -1\right)$$

(28) $$W_{I}=-\gamma^{\mathrm{SM}}=\sigma^{\mathrm{SV}}-\sigma^{\mathrm{SM}}=\sigma^{\mathrm{VM}}\cos\theta$$

where *σ*^VM^ ≡ *γ*^VM^ is the surface tension, (*σ*^SV^ *– σ*^SM^) ≡ –*γ*^SM^ is the adhesion tension [62], and *θ* the contact angle of the corresponding solid–fluid–vapor interface.

Table 2 illustrates the wetting functions and contact angles of the PS/SiO_2_ interphases studied here for *N*_m_ = 384; they remain practically the same with larger chain lengths as shown in Figure S4. *W*_A_ corresponds to the reversible work required to separate two phases in contact. It is noted that in the absence of the ramp potential, *W*_A_ is significantly lower than in the HW and PW cases, where *v*_ramp_ has been fitted to experimentally reported values of *W*_A_. *W*_S_ quantifies the spontaneity of the wetting process: positive values indicate spontaneous spreading across the interphase (perfect wetting), while negative values indicate finite contact angles (partial or no wetting). In the LW and HW interfaces, *W*_S_ remains negative across the full molar mass regime investigated here, indicating that the corresponding solid–fluid–vapor interphase will form finite contact angles. The PW interface, on the other hand, exhibits positive *W*_S_; thus, PS will spread spontaneously on the silica surface.

#### 4.1.2. Grafted/Vacuum (GV) and Grafted/Melt (GM) Interphases

**Figure 3:**
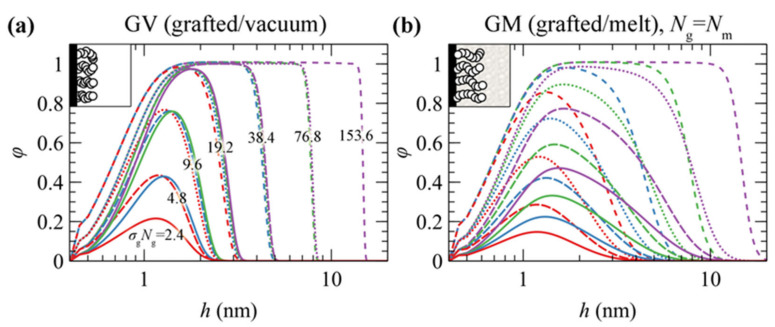
Reduced density profiles of polystyrene PS brushes from SL-SGT EoS in the absence of the ramp potential in (**a**) grafted/vacuum (GV) and (**b**) grafted/melt (GM) systems for *N*_g_ = 24 (red), 48 (blue), 96 (green) and 192 (violet), and *σ*_g_ = 0.1 (solid lines), 0.2 (dashes), 0.4 (dots) and 0.8 (short dashes) nm^–2^. The numbers in (**a**) depict the value of the product *σ*_g_*Ν*_g_ in nm^–2^ units.

Figure 3 depicts the reduced density profiles of PS grafted chains in (a) GV and (b) GM systems in the absence of the ramp potential (low wetting) as a function of *σ*_g_ and *Ν*_g_. 

The behavior of grafted chains can be classified into three distinct regimes depending on the combinations of *σ*_g_ and *N*_g_:

1. Mushroom regime (low *σ*_g_*N*_g_). The density of the profiles is less than the bulk density and chains assume randomly coiled conformations [63]. Increasing *σ*_g_ and *N*_g_ has a minor effect on the thickness of the profiles but rather makes them more pronounced.
2. Dense brush regime (high *σ*_g_*N*_g_). The brushes become stretched [46,63] and feature extended regions with bulk density. The thickness of the profiles depends strongly on both *σ*_g_ and *N*_g_, reaching the limiting scaling behavior ~*σ*_g_^1^*N*_g_^1^; i.e., the dimensions of the brushes become proportional to their mass.
3. Crowding regime (very high *σ*_g_). In this regime, the crowding of the chains becomes so intense that the density of the grafted chains surpasses slightly the bulk one, as observed in our previous work [46]. This happens because the entropic penalty due to stretching overcomes the enthalpic penalty due to deviations from the bulk density.

There are, however, noticeable differences between the two systems. In GV, the thickness of the density profiles becomes commensurate with the number of the grafted PS segments which equals the product *σ*_g_*Ν*_g_. Indeed, the profiles in Figure 3a collapse together for constant *σ*_g_*Ν*_g_ values, i.e., for constant amount of material. In GV, the tails of the profiles feature a sigmoid region on the order of 1 nm at the polymer/vacuum interface [44,61], whereas in GM they are much more expanded across the bulk region [15,46]. Finally, in both GV and GM, the profiles become slightly more pronounced with increasing *σ*_g_ in the vicinity of the grafting points (~0.4 nm).

The dimensions of the grafted chains can be quantified in terms of their root mean squared brush thickness, which is calculated via the following Equation (29).

(29) $${\langle h_{g}{}^{2}\rangle }^{1/2}={\left[\frac{\int_{R}^{}dr\;{\left[h\left(r\right)\right]}^{2}\rho_{g}(r)}{\int_{R}^{}dr\rho_{g}(r)}\right]}^{1/2}$$

Additionally, we employ another measure for the extension of grafted chains, namely the height, $h_{99\%}$, which is defined here as the distance between the surface of the solid ($\partial R_{\mathrm{solid}}^{}$) and a surface ($\partial R_{h_{99\%}}^{}$) that encloses 99% of grafted chain segments. It is determined via the following Equation (30).

(30) $$\int_{R_{99\%}^{}}^{}dr\rho_{g}(r)=0.99N_{g}n_{g}$$

with $R_{99\%}^{}$ denoting the region between $\partial R_{\mathrm{solid}}^{}$ and $\partial R_{h_{99\%}}^{}$.

**Figure 4:**
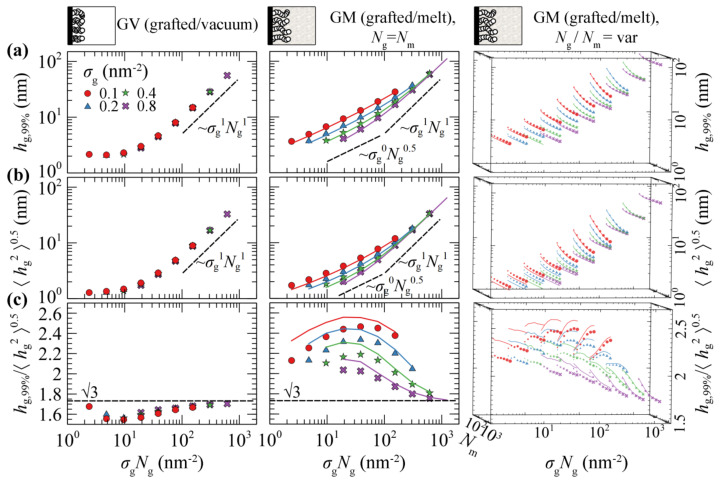
(**a**) $h_{g}{}_{,99\%}$, (**b**) ${\langle h_{g}{}^{2}\rangle }^{0.5}$, and (**c**) their ratio $h_{g}{}_{,99\%}/{\langle h_{g}{}^{2}\rangle }^{0.5}$ versus the scaling law *σ*_g_*N*_g_ using the SL-SGT (markers) and HFD (solid lines) EoS. The panels on the left column correspond to the GV system. The panels in the central row depict results regarding the GM system, for *N*_g_ = *N*_m_. The rightmost column depicts results for the GM system as well, but with *N*_m_ varying from 24 up to 1536 segments. Different colors and symbols denote different values of the surface grafting density; *σ*_g_ = 0.1 (red/circles), 0.2 (blue/triangles), 0.4 (green/stars) and 0.8 (violet/crosses) nm^–2^. The length of grafted chains increases implicitly in each panel from left to right, according to the scaling law expression presented in the labels of the *x*-axis. In the rightmost column the size of the symbols increases with the chain length of matrix chains. All cases have been evaluated in the absence of the ramp potential.

Figure 4a,b illustrates evaluations of ${\langle h_{g}{}^{2}\rangle }^{1/2}$ and $h_{99\%}$ against the scaling law ~*σ*_g_*N*_g_ in GV (left column) and GM with the matrix chains either being equal in length to the grafted ones (*N*_m_ = *N*_g_; central column), or varying between 24 and 1536 segments (right column). Figure 4c depicts the ratio $h_{99\%}/{\langle h_{g}{}^{2}\rangle }^{1/2}$ which can be thought of as a measure of the shape of the profile. A striking difference between the two systems is that in the first one, the measures of the brush thickness collapse to a single master curve across the full regime. This is because in GV, the shape of the collapsed films is a function of the mass of the film (~*σ*_g_*N*_g_) and does not depend on the ratio (*σ*_g_/*N*_g_); see Figure 3a.

Across the mushroom regime, the brush thickness is practically independent of *σ*_g_. The thickness in vacuum is independent of *N*_g_ as well, indicating collapse of the sparsely grafted chains on the surface. In the case where melt chains are present, the thickness exhibits a random walk-like *N*_g_-dependence, scaling approximately as ~*N*_g_^0.5^. The shape of the brushes as quantified by the ratio $h_{99\%}/{\langle h_{g}{}^{2}\rangle }^{1/2}$, is quite sensitive to the grafting density in the presence of matrix chains; in GV, the ratio decreases with respect to the predictions from Alexander’s model [64,65] for incompressible brushes, whilst the opposite behavior is exhibited in the GM system.

Across the dense brush regime the brush dimensions depend strongly on both *σ*_g_ and *N*_g_. For very large *σ*_g_ and *N*_g_ the brush thickness scales as ~*σ*_g_*N*_g_, no matter the solvent conditions (vacuum or melt). This means that the dimensions of the brushes become proportional to their mass and the ratio $h_{99\%}/{\langle h_{g}{}^{2}\rangle }^{1/2}$ approaches the limiting value of $\sqrt{3}$ predicted by Alexander’s model [64,65].

Regarding the effect of the matrix chains on the scaling of the brushes, it appears that, as long as $N_{g}\leq N_{m}$, the brush dimensions are practically independent of *N*_m_. For $N_{m}\ll N_{g}$, on the other hand, the brushes expand with decreasing *N*_m_ due to the fact that the matrix chains can easily penetrate the brushes, thus the latter swell towards the bulk region. A similar behavior has been recently observed by Biltchak et al. [39]. Therefore, modulating *N*_m_ allows for the tuning of the solvent conditions, from theta solvent (*N*_m_ = *N*_g_) up to good solvent (*N*_m_ < *N*_g_) conditions. Regarding the ratio $h_{99\%}/{\langle h_{g}{}^{2}\rangle }^{1/2}$, it features a complicated behavior with varying *N*_m_, where it decreases/increases for low/high *σ*_g_.

It seems that, regardless of the choice of the free energy density equation (e.g., compare lines (HFD) with markers (SL-SGT) in Figure 4) or the strength of the polymer–solid interactions (see Figure S5), the structural features and the scaling behaviors of GM systems are quantitatively very similar. In addition, the shortcoming of using HFD with constant *κ*_T_ does not have a practical effect on the structural properties of grafted chains and on the potential of mean force of the system, as will be shown below.

**Figure 5:**
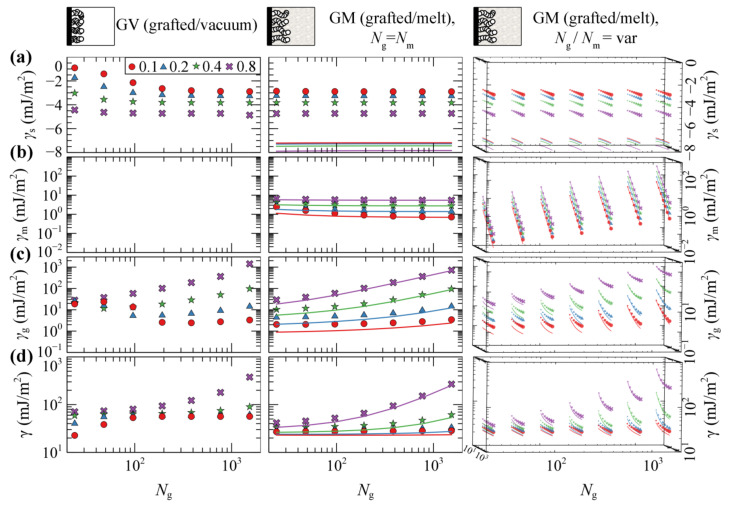
Partial contributions to the grand potential (**a**) γ_s_, (**b**) *γ*_m_, and (**c**) *γ*_g_, and (**d**) total grand potential, *γ*, per unit area, using the SL-SGT (markers) and HFD (solid lines) EoS. The panels on the left column correspond to the GV system. The panels in the central column depict results regarding the GM system, for *N*_g_ = *N*_m_. The rightmost column depicts results for the GM system as well, but with *N*_m_ varying from 24 up to 1536 segments. Different colors and symbols denote different values of the surface grafting density; *σ*_g_ = 0.1 (red/circles), 0.2 (blue/triangles), 0.4 (green/stars) and 0.8 (violet/crosses) nm^–2^. The chain length of grafted chains increases implicitly in each panel from left to right, according to the scaling law expression presented in the labels of the *x*-axis. In the rightmost column the size of the symbols increases with the chain length of matrix chains. All cases have been evaluated in the absence of the ramp potential.

Figure 5 illustrates the total grand potential per unit area of grafted/vacuum ($\gamma_{}^{\mathrm{GV}}$) and grafted/melt ($\gamma_{}^{\mathrm{GM}}$) interphases as a function of *N*_g_ and *σ*_g_, as well as the partial contributions from the polymer/solid interactions ($\gamma_{s}^{}$), and the entropy of matrix ($\gamma_{m}^{}$) and grafted chains ($\gamma_{g}^{}$). Some key remarks regarding the evaluation of each term with SL-SGT can be summarized as follows:

- $\gamma_{s}^{}$ is purely of enthalpic origin and thus it is a functional of the total density profiles. $\gamma_{s}^{}$ becomes more attractive with increasing *σ*_g_, since the profiles become more pronounced in the vicinity of the solid (e.g., see Figure 3). In GV, $\gamma_{s}^{\mathrm{GV}}$ decreases with increasing *N*_g_, since increasing *N*_g_ increases the amount of material near the solid. In GM,$\gamma_{s}^{\mathrm{GM}}$ is independent of *N*_g_, since the total density profiles are also invariant to *N*_g_, e.g., compare profiles in Figure 2c–f for different chain lengths.
- $\gamma_{m}^{}$ describes the entropic contribution of the matrix chains. In GM, it decreases precipitously with increasing $N_{g}/N_{m}$ ratio, since the grafted chains occupy more space in the vicinity of the Interphase leaving the matrix chains with fewer available conformations. Note that, by keeping *N*_g_ fixed, *γ*_m_ scales with *N*_m_ about as *γ*_m_ ~ *N*_m_^−1^; see Equation (8). In GV systems, this term is of course zero since there are no matrix chains at all.
- $\gamma_{g}^{}$ quantifies the entropic contribution of the grafted chains and it is an *indicator* of the stretching of the brush [46]. It increases monotonically with increasing *N*_g_ and *σ*_g_, since the grafted chains expand and stretch further towards the bulk region. In addition, $\gamma_{g}^{\mathrm{GM}}$ increases with decreasing *N*_m_, since the grafted chain-melt interactions are enhanced and, as a result, the brushes swell as shown in Figure 4b,c (rightmost column).
- γ is the total free energy of the interfacial systems per unit area. It increases with increasing molecular weight of grafted chains and appears to be dominated by the conformational entropy term of grafted chains, $\gamma_{g}^{}$.

Overall, the evaluations of the free energy terms with HFD are in good qualitative agreement with SL-SGT. Furthermore, it appears that evaluating $\gamma_{s}^{\mathrm{GM}}$ with HFD is more negative than when the SL-SGT EoS is used, because the density profiles lie closer to the silica surface in the former model (e.g., compare Figure 2d,f). In their recent work concerning neat grafted nanoparticles, Mydia et al. [40] report that for constant grafting density, the stretching energy does not increase monotonically with the chain length. This behavior is attributed to the curved space around the nanoparticles since, at some point, the grafted chains do not experience the presence of each other and become unperturbed. For planar surfaces, however, the threshold chain length becomes infinitely large, since no curvature is involved and the chains will always experience the presence of each other, considering that the dimensionless quantity *σ*_g_*R*_g_^2^ is above a threshold value as well [46].

### 4.2. PS Melt Capped between Two Bare Silica Surfaces (SMS)

It is worth studying the potential of mean force (PMF) between two approaching bare silica surfaces. This means that only matrix chains are present in the system. This situation corresponds to the limiting case of very low grafting densities, where allophobic dewetting occurs and the enthalpic interactions between the two solid surfaces prevail. The matrix chains are gradually restricted in terms of available conformations as the distance between the two plates decreases. For these calculations, the PMF^SMS^ is expressed with respect to the free energies of the single SM interphases for the same chain length:

(31) $${\mathrm{PMF}}^{\mathrm{SMS}}=\gamma^{\mathrm{SMS}}-\gamma_{\inf}^{\mathrm{SMS}}=\gamma^{\mathrm{SMS}}-\underset{h_{\mathrm{ss}}\to \infty }{\lim}\gamma^{\mathrm{SMS}}=\gamma^{\mathrm{SMS}}-2\gamma^{\mathrm{SM}}$$

with $\gamma^{\mathrm{SM}}$ being the free energy of a SM interphase in presence of matrix chains of length $N_{m}$, depicted in Figure 2a,b. Note that, with PMF^SMS^ known, the disjoining pressure can be calculated as:

(32) $$\Pi \left(h_{\mathrm{ss}}\right)=-{\left(\frac{\partial \gamma^{\mathrm{SMS}}}{\partial h_{\mathrm{ss}}}\right)}_{\mu ,T}$$

Due to numerical stability issues, the initial configuration of the field can affect the outcome of the converged solution. To investigate this effect, we performed these calculations using two different compression methods:

1. In the first method, the calculations were performed in a decremental fashion, in which the initial configuration of the field was set to the field corresponding to the converged calculation for a slightly larger domain, ${w^{\prime }}_{\mathrm{ifc},\mathrm{init}}\left(h_{\mathrm{ss}}\right)={w^{\prime }}_{\mathrm{ifc},\mathrm{final}}\left(h_{\mathrm{ss}}+\Delta h\right)$
2. In the calculations corresponding to the second method, the initial configuration of the field was set to zero across the domain, ${w^{\prime }}_{\mathrm{ifc},\mathrm{init}}=0$.

Using the first method, it is easier to derive a solution that corresponds to a stable configuration. The second method, on the other hand, can provide a measure of the stability of the films in terms of their tendency to collapse and their sensitivity to fluctuations about equilibrium (e.g., their response during the formation of a cavity).

**Figure 6:**
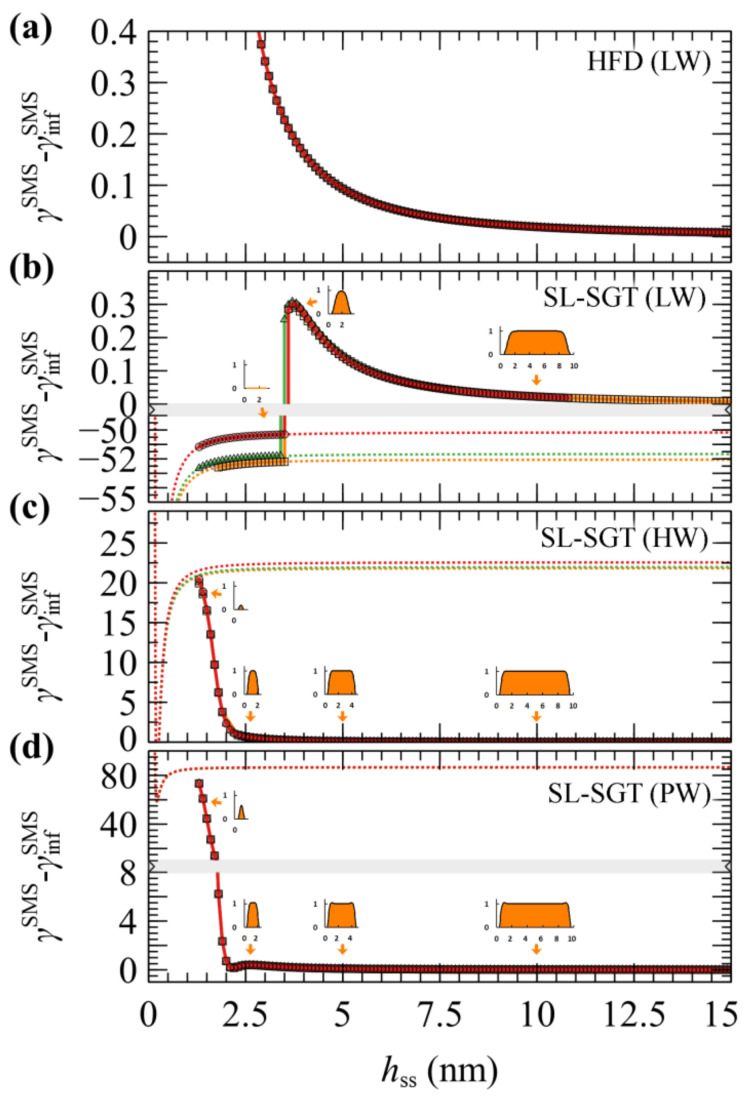
Potential of mean force, in mJ/m^2^, for the system of approaching bare silica surfaces in a melt (SMS) obtained by the (**a**) HFD, (**b**) SL-SGT (LW), (**c**) SL-SGT (HW) and (**d**) SL-SGT (PW), for *N*_m_ = 24 (red), 96 (green), and 384 (orange). The calculations were performed in a decremental fashion, in which ${w^{\prime }}_{\mathrm{ifc},\mathrm{init}}\left(h_{\mathrm{ss}}\right)={w^{\prime }}_{\mathrm{ifc},\mathrm{final}}\left(h_{\mathrm{ss}}+\Delta h\right)$, with a compression rate equal to −0.1 nm/evaluation. The inset graphs in (b-d) depict snapshots of the density profiles at plate-plate distances denoted by the arrows, for *N*_m_ = 384. Bands denote scale changes along the axes. The dashed lines display the Hamaker potential contribution to the solid–solid interaction, shifted by twice the solid/polymer adhesion tension.

Figure 6 illustrates evaluations of PMF^SMS^ with HFD (a), and with SL-SGT under low (b, LW), high (c, HW) and perfect (d, PW) wetting situations, using the first method for decremental compression. Results concerning the second method are presented in Supplementary Materials Section S5, along with a relevant discussion.

In the case of HFD, regardless of the compression approach, PMF^SMS^ increases with decreasing plate-plate distance, suggesting the manifestation of a repulsive force that resists the attractive interactions between the surfaces. These repulsive forces are dominated by the loss of polymer–solid interactions; with decreasing *h*_ss_, the mass of the film decreases and there are fewer interaction sites. The sign of these forces depends on an interplay between the strength of the polymer–solid and the solid–solid interactions. If the latter become much stronger than the former, the solid–solid forces will dominate and PMF^SMS^ will become attractive. The steadily increasing forces in this case can be, however, a misleading result, because HFD does not account for the gaseous phases which may arise during the process.

According to evaluations with SL-SGT under low wetting (LW) conditions (Figure 6b), the functional dependence of PMF^SMS^ is quite similar to that of HFD. However, below a critical plate-plate distance ($h_{\mathrm{ss}}^{\mathrm{crit}}$), PMF^SMS^ decreases abruptly, indicating a phase transition. At these distances it is impossible for SCF to maintain a metastable film; hence, a cavity is formed and the calculation converges to the more stable solution, **φ** = **0**. The films remain stable above $h_{\mathrm{ss}}^{\mathrm{crit}}$ on the order of 3.5 nm, regardless of *N*_m_ (Figure 6b).

Upon departure of the melt from the gap between the plates, the only contribution to the free energy is due to plate-plate interactions which are described here by means of the Hamaker potential; therefore, leading to the eventual contact of the adjacent solid surfaces. Note that, for the LW surfaces, ${\mathrm{PMF}}^{\mathrm{SMS}}\left(\varphi =0\right)=\gamma_{\mathrm{ss}}^{\mathrm{SMS}}-\gamma_{\inf}^{\mathrm{SMS}}$, which is depicted by dashed lines in Figure 6b is negative, indicating that the polymer–solid interactions are really weak for the LW films and these films are actually metastable with respect to cavitation across the entire range of thicknesses.

A similar picture has been reported by past simulations from a variable-density lattice based SCF model [66]. In that model, the interactions in flat geometries become insignificant for *h*_ss_ slightly larger or equal than $4{\langle R_{g}{}^{2}\rangle }^{0.5}$, whereas the maximum recorded force per radius before cavitation in a crossed cylinder geometry was on the order of ~0.01 mN/m when considering high energy surfaces and 0.1 mN/m for low energy surfaces.

With enhanced polymer/solid interactions, the stability of the capped polymer films is reinforced considerably. According to the more reliable solution scheme where the domain length is adjusted decrementally, the HW and PW films remain stable throughout the full *h*_ss_ range examined here, always converging to the stable, polymer-filled equilibrium solution of the problem (see Figure 6c,d). In contrast to LW films, the PMF^SMS^ of the HW and PW ones increases steadily for *h*_ss_ less than 2.5 nm, whereas in the limit of low *h*_ss_ the density decreases significantly due to the entropy of confinement. A significant free energy barrier of approximately −$\gamma_{\inf}^{\mathrm{SMS}}$, on the order of 20 mN/m in the HW case and 80 mN/m in the PW case, has to be overcome for the polymer to be expelled completely and the solids to come in direct contact at ${\mathrm{PMF}}^{\mathrm{SMS}}\left(\varphi =0\right)=\gamma_{\mathrm{ss}}^{\mathrm{SMS}}-\gamma_{\inf}^{\mathrm{SMS}}$.

There is, however, a striking difference between the HW and PW films. The HW films can be considered as metastable with respect to cavitation, since, after crossing a barrier of ~−$\gamma_{\inf}^{\mathrm{SMS}}$ with decreasing *h*_ss_, they can be trapped in the global minimum of attractive Hamaker interactions between the bare solids (see minima of the dashed lines in 6c), as in the case of the LW film. The PW film, on the other hand, is indeed stable down to thicknesses of ca. 1.5 nm, since the minimum of the Hamaker potential lies way higher (~+60 mJ/m^2^) than the plateau PMF^SMS^ value at large *h*_ss_.

### 4.3. Interacting Grafted Surfaces in Melt (GMG)

The current section presents evaluations of the potential of mean force of approaching grafted surfaces. To facilitate comparisons across the wide parameter space considered in this work, the PMF^GMG^ will be expressed in terms of the reduced surface-surface distance which is defined by the following Equation (33).

(33) $${\tilde{h}}_{\mathrm{ss}}=\frac{2h_{\mathrm{ss}}}{{\langle h_{g^{-}}{}^{2}\rangle }^{0.5}+{\langle h_{g^{+}}{}^{2}\rangle }^{0.5}}$$

with ${\langle h_{g^{-}}{}^{2}\rangle }^{0.5}$ and ${\langle h_{g^{+}}{}^{2}\rangle }^{0.5}$ being the root mean squared brush thickness of the single grafted surfaces (infinite surface-surface distance) at the same temperature and in the presence of matrix chains of length $N_{m}$ (brush thickness from Figure 4). A similar normalization can be obtained by dividing $h_{\mathrm{ss}}$ with ${\langle R_{g}{}^{2}\rangle }^{0.5}$, since ${\langle R_{g}{}^{2}\rangle }^{0.5}\sim N_{g}$ and $N_{g}\sim {\langle h_{g}{}^{2}\rangle }^{0.5}$ across the dense brush regime. However, normalizing the plate-plate separation distances as shown in Equation (33) is a more natural approach of making such comparisons, since it allows evaluating the tendencies of the brushes to interpenetrate. In addition,${\langle h_{g}{}^{2}\rangle }^{0.5}$ takes account of the chain perturbations when varying the melt conditions and leads to comparisons that are less sensitive to the particular equation of state used in the nonbonded free energy density model.

As in the SMS systems, PMF^GMG^ will be expressed relative to the free energy of the isolated $G^{-}M$ and ${\mathrm{GM}}^{+}$ systems in the presence of matrix chains of length *N*_m_ (see Figure 5).

(34) $${\mathrm{PMF}}^{\mathrm{GMG}}=\gamma^{\mathrm{GMG}}-\gamma_{\inf}^{\mathrm{GMG}}=\gamma^{\mathrm{GMG}}-\underset{h_{\mathrm{ss}}\to \infty }{\lim}\gamma^{\mathrm{GMG}}=\gamma^{\mathrm{GMG}}-\gamma^{G^{-}M}-\gamma^{{\mathrm{MG}}^{+}}$$

In other words, PMF^GMG^ is expressed with respect to the free energy of the system at infinite plate-plate separation. Regarding the segment/substrate interactions—unless otherwise stated—LW conditions are employed throughout this section.

#### 4.3.1. Symmetric Surfaces

**Figure 7:**
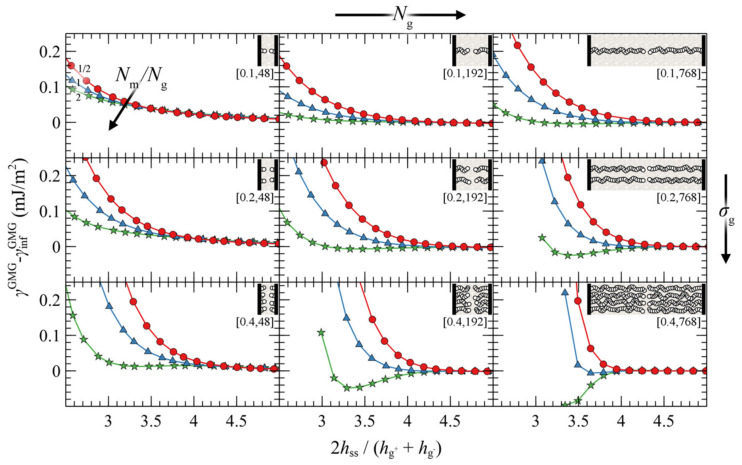
Potential of mean force against the reduced surface-surface distance in a symmetric system of approaching grafted silica surfaces in a melt from the SL EoS. Colors correspond to evaluations for $N_{m}/N_{g}$ = 1/2 (red), 1 (blue) and 2 (green), whereas the labels in brackets denote *σ*_g_ (nm^−2^) and *N*_g_. Lines are guides to the eye.

**Figure 8:**
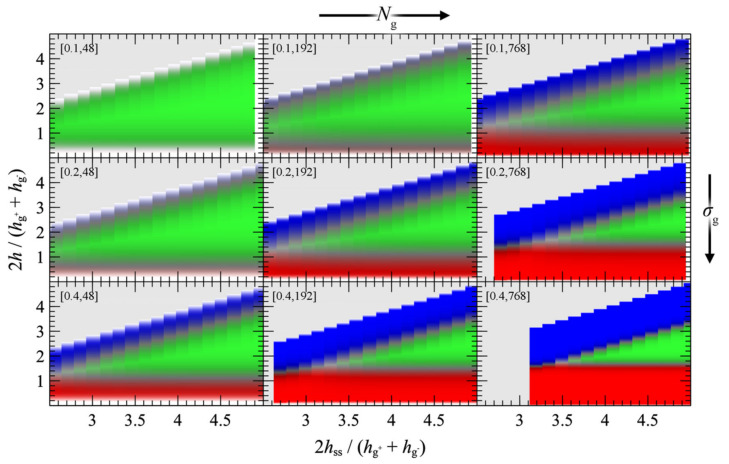
Reduced density distributions corresponding to the PMF^GMG^ panels in Figure 7**,** as a function of the plate-plate distance (abscissa) and the distance from the left wall (ordinate) in reduced units. Red/green/blue colors correspond to regions with high density (*φ_c_* = 1) of *c* = g^−^/m/g^+^ chains, respectively. Grey denotes regions which lie outside the modeled domain or have not been evaluated at all. Labels in brackets denote *σ*_g_ (nm^−2^) and *N*_g_.

We present the PMF^GMG^ for the simplest case where the opposing surfaces are grafted symmetrically with respect to *σ*_g_ and *N*_g_ for varying *N*_m_/*N*_g_. Figure 7 illustrates PMF^GMG^ as a function of the plate-plate distance, *h*_ss_, whereas Figure 8 depicts the corresponding density distribution across the examined parameter space for *N*_m_ = *N*_g_.

According to Figure 7, the brushes start to experience the presence of each other at distances in the order of 4–5 ${\tilde{h}}_{\mathrm{ss}}$, while for larger ${\tilde{h}}_{\mathrm{ss}}$, PMF^GMG^ ≃ 0. At lower separation distances the brushes interact strongly with each other and PMF^GMG^ increases. In systems with low *σ*_g_ (mushroom regime), the brushes are relatively soft and thus PMF increases at lower ${\tilde{h}}_{\mathrm{ss}}$. In dense systems, on the other hand, the brushes are more compact and as a result the PMF increases abruptly at larger ${\tilde{h}}_{\mathrm{ss}}$, on the order of 3–3.5 (see bottom right panel of Figure 7). This regime coheres with the predictions of Alexander’s model for incompressible brushes [64,65], where, regardless of *N*_g_ and *σ*_g_, the predicted separation distance of two Alexander brushes in contact is ${\tilde{h}}_{\mathrm{ss},\min}=2\sqrt{3}\sim 3.4$; the calculation is illustrated in Appendix A.

Regarding the effect of varying the chain length of matrix chains on PMF^GMG^, for $N_{m}/N_{g}$ ≤ 1, PMF^GMG^ becomes strictly repulsive, with the exception of the case for densely grafted and long grafted chains, *σ*_g_ = 0.4 nm^2^, *N*_g_ = 768. Increasing $N_{m}/N_{g}$ leads to the manifestation of attractive interactions as indicated by the formation of a minimum in PMF^GMG^ (autophobic dewetting). These interactions become slightly stronger with increasing *N*_g_, and even stronger with increasing *σ*_g_. The enhancement of the attractive interactions with increasing grafting density has been also observed in several theoretical [22,25,67] and experimental studies [68,69,70,71,72].

**Figure 9:**
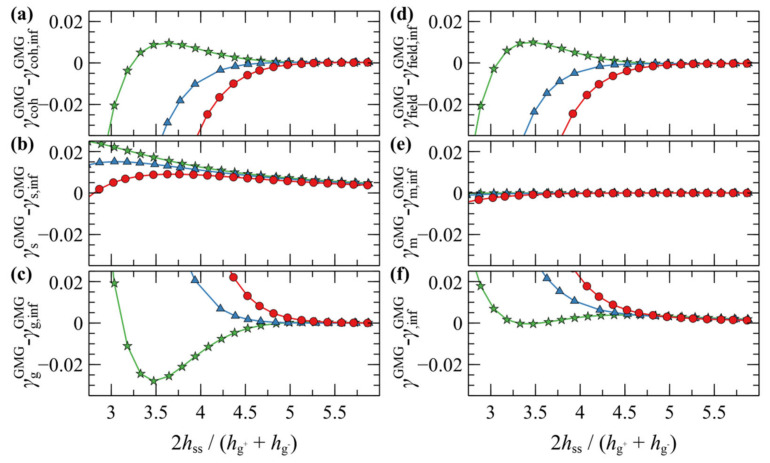
Free energy partial contributions to the potential of mean force, in mJ/m^2^, of two approaching symmetric grafted surfaces with *σ*_g_ = 0.2 nm^–2^ and *N*_g_ = 192 embedded in a melt with *N*_m_/*N*_g_ = 0.5 (circles), 1 (triangles) and 2 (stars): (**a**) cohesive interactions, (**b**) polymer-solid interactions, (**c**) entropic contribution from grafted chains, (**d**) density-field interactions, (**e**) entropic contributions of matrix chains and (**f**) total grand potential.

Figure 9 illustrates the contribution of individual free energy terms to PMF^GMG^ for a case with *σ*_g_ = 0.2 nm^–2^ and *N*_g_ = 192. It appears that the attractive part of PMF^GMG^ is dominated by the entropic contribution of the grafted chains, $\gamma_{g}^{\mathrm{GMG}}$, shown in Figure 9c. In addition, the terms responsible for the cohesive and the chemical potential-density field interactions change due to minor variations in the mean density profile and exhibit an opposite trend to that of $\gamma_{g}^{\mathrm{GMG}}$, albeit weaker. That PMF^GMG^ becomes more repulsive when *N*_m_ < *N*_g_ is attributed to the matrix chains being able to penetrate the space occupied by the brush, compelling the grafted chains of the two surfaces to expand in space until they interact with each other, thus keeping the two surfaces separated. In other words, the solvent conditions improve with decreasing *N*_m_/*N*_g_; hence, the brushes prefer to interact with the solvent molecules than with each other.

In general, the evolution of the term associated with the polymer–solid interactions, $\gamma_{s}^{\mathrm{GMG}}$, with decreasing plate-plate distance, depends on an interplay between two processes: (i) The thinner the polymer film becomes, the fewer polymer segments can interact with the solid, thus leading to increased $\gamma_{s}^{\mathrm{GMG}}$ (less attractive). (ii) The brushes can become slightly denser with decreasing ${\tilde{h}}_{\mathrm{ss}}$, hence leading to decreased $\gamma_{s}^{\mathrm{GMG}}$ (more attractive). Indeed, for $N_{m}>N_{g}$, $\gamma_{s}^{\mathrm{GMG}}$ is dominated by the first process, since it increases monotonically with decreasing ${\tilde{h}}_{\mathrm{ss}}$. However, for $N_{m}<N_{g}$ it features an interesting behavior where it initially increases with decreasing ${\tilde{h}}_{\mathrm{ss}}$, while for ${\tilde{h}}_{\mathrm{ss}}<3.8$ it decreases, indicating that the second process dominates.

#### 4.3.2. Asymmetric Surfaces

**Figure 10:**
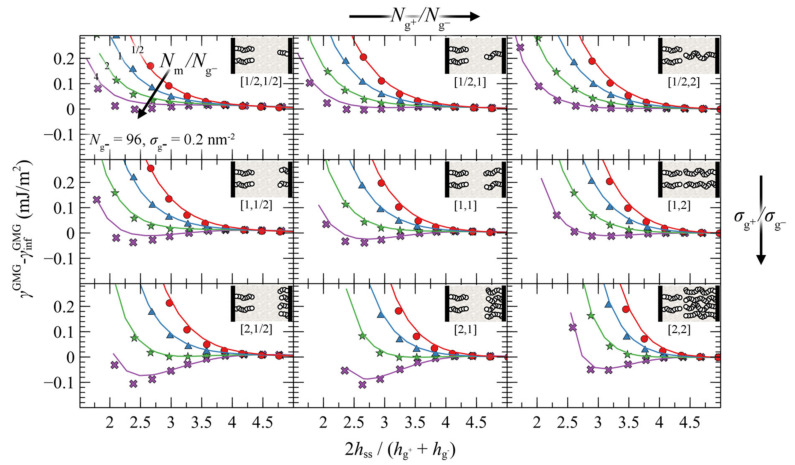
Potential of mean force against the reduced surface-surface distance for approaching grafted silica surfaces with $\sigma_{g^{-}}$ = 0.2 nm^−2^ and $N_{g^{-}}$ = 96. Colors correspond to evaluations for $N_{m}/N_{g^{-}}$ = 1/2 (red), 1 (blue), 2 (green) and 4 (purple), and the labels in brackets denote the ratios $\sigma_{g^{+}}/\sigma_{g^{-}}$ and $N_{g^{+}}/N_{g^{-}}$. Lines and markers correspond to evaluations with the HFD and SL EoS, respectively. Inset: each bead denotes a segment of 24 PS monomers.

The present section investigates the effect of asymmetry of the opposing grafted surfaces in terms of the relative chain lengths and grafting densities. In particular, Figure 10 illustrates evaluations of PMF^GMG^ for constant chain length and grafting density at the lower face ($N_{g^{-}}$ = 96, $\sigma_{g^{-}}$ = 0.2 nm^−2^) and varying $N_{g^{+}}/N_{g^{-}}$, $N_{m}/N_{g^{-}}$ and $\sigma_{g^{+}}/\sigma_{g^{-}}$. Similarly, Figure S7 and Figure S8 depict the same evaluations but for four times larger $N_{g^{-}}$ and for two times larger $\sigma_{g^{-}}$, respectively.

Irrespectively of the degree of asymmetry, adjacent brushes experience the presence of each other at distances commensurate with 4–5 ${\tilde{h}}_{\mathrm{ss}}$, similarly to the symmetric case in Figure 7. By keeping $\sigma_{g^{-}}$, $N_{g^{-}}$ constant, the ratio $N_{m}/N_{g^{-}}$ fixed at various values (same colors), and varying $\sigma_{g^{+}}$ and $N_{g^{+}}$, some general trends are emerging: the attractive interactions between the surfaces become stronger with increasing $\sigma_{g^{+}}/\sigma_{g^{-}}$ ratios (from top to bottom) and with decreasing $N_{g^{+}}/N_{g^{-}}$ (from right to left).

That PMF^GMG^ becomes more attractive with increasing $\sigma_{g^{+}}/\sigma_{g^{-}}$ is to be expected: upon increasing $\sigma_{g^{+}}$ the mean grafting density increases as well, thus PMF^GMG^ becomes more attractive, as in the case for the symmetric surfaces in Figure 7. However, that the interactions become more attractive with decreasing $N_{g^{+}}/N_{g^{-}}$ has to be reconciled with the findings reported in Figure 7 for symmetrically grafted surfaces. To interpret this effect, one should take into account that during these evaluations the ratio $N_{m}/N_{g^{-}}$ was fixed, whereas varying $\sigma_{g^{+}}$ and $N_{g^{+}}$ can have direct implications for the effective ratio of *N*_m_ with respect to the average size of grafted chains.

Let $N_{\bar{g}}$ be the average chain length of grafted chains, estimated as the weighted average of grafted chain length with respect to the grafting densities:

(35) $$N_{\bar{g}}=\frac{\sigma_{g^{-}}N_{g^{-}}+\sigma_{g^{+}}N_{g^{+}}}{\sigma_{g^{-}}+\sigma_{g^{+}}}$$

$N_{m}/N_{\bar{g}}$ is a measure of the length of matrix chains in relation to grafted chains. Based on Equation (35) it is evident that, upon decreasing $N_{g^{+}}/N_{g^{-}}$ at constant $N_{g^{-}}$ and $\sigma_{g^{+}}/\sigma_{g^{-}}$, $N_{\bar{g}}$ decreases as well and thus the effective ratio $N_{m}/N_{\bar{g}}$ increases. Increasing the size of matrix chains was shown to enhance the attractive interactions in Figure 7, and the results shown in Figure 10 are consistent with this trend.

**Figure 11:**
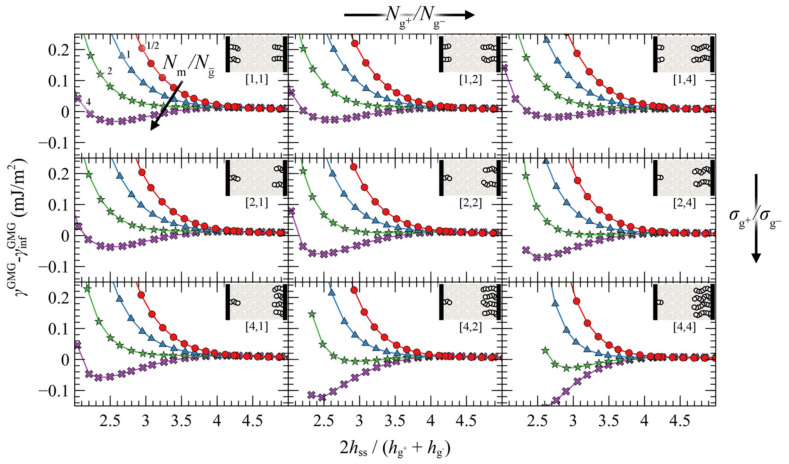
Potential of mean force against the reduced surface-surface distance for approaching grafted silica surfaces. Colors correspond to evaluations for $N_{m}/N_{\bar{g}}$ = 1/2 (red), 1 (blue), 2 (green) and 4 (purple), whereas the small legends in brackets below the insets denote the ratios $\sigma_{g^{+}}/\sigma_{g^{-}}$ and $N_{g^{+}}/N_{g^{-}}$, respectively. The top left panel corresponds to the reference symmetric case with $\sigma_{g}^{\mathrm{ref}}=\sigma_{g^{-}}=\sigma_{g^{+}}$ = 0.2 nm^–2^ and $N_{g}^{\mathrm{ref}}=N_{g^{-}}=N_{g^{+}}$ = 96. The actual $N_{g^{\pm }}$ and $\sigma_{g^{\pm }}$ for each case can be retrieved as follows: $N_{g^{\pm }}=N_{g}^{\mathrm{ref}}n^{\pm 1/2}$ and $\sigma_{g^{\pm }}=\sigma_{g}^{\mathrm{ref}}m^{\pm 1/2}$, with [*n*, *m*] = [$N_{g^{+}}$/$N_{g^{+}}$,$\sigma_{g^{+}}$/$\sigma_{g^{-}}$] being the numbers at the legends under the insets. It should be noted that inset schematics belonging to panels other than the corner ones are only approximate; in these cases, $\sigma_{g^{+/-}}$ and $N_{g^{+/-}}$ are scaled by a factor of $\pm \sqrt{2}$.

An alternative way to interpret and isolate the effect of asymmetry is to vary the ratios $\sigma_{g^{+}}/\sigma_{g^{-}}$ and $N_{g^{+}}/N_{g^{-}}$, but fix the effective ratio $N_{m}/N_{\bar{g}}$ based on Equation (35). Figure 11 depicts such evaluations, wherein the top/left panel depicts the reference symmetric case (*σ*_g_ = 0.2 nm^–2^, *N*_g_ = 96), while the asymmetry with respect to $\sigma_{g}$ ($N_{g}$) increases from top-to-bottom (left-to-right). As can be seen, varying $\sigma_{g^{+}}/\sigma_{g^{-}}$ or $N_{g^{+}}/N_{g^{-}}$ individually has a minor effect on PMF^GMG^. This finding is important, since it shows that minor deviations in $\sigma_{g}$ and $N_{g}$ do not affect PMF^GMG^ provided $N_{m}/N_{\bar{g}}$ is fixed. On the other hand, PMF^GMG^ can become very attractive in extreme cases of asymmetry where both the asymmetry in *σ*_g_ and *N*_g_ increases (e.g., see bottom right panel in Figure 11), and this is mainly attributed to the increased average grafting density.

#### 4.3.3. Surface Energy

**Figure 12:**
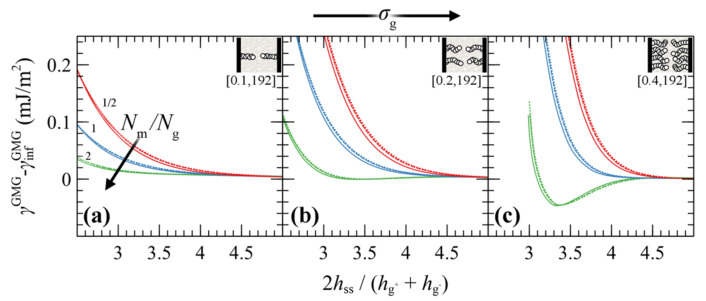
Potential of mean force against the reduced surface-surface distance of approaching symmetric grafted silica surfaces in a melt from the SL EoS for *σ*_g_ set to (**a**) 0.1, (**b**) 0.2 and (**c**) 0.4 nm^–2^ and *N*_g_ = 192. Colors correspond to evaluations for $N_{m}/N_{g}$ = 1/2 (red), 1 (blue) and 2 (green), whereas different styles denote interfaces with low (solid lines), high (dashed lines) and perfect (dots) wetting.

Figure 12 presents the PMF^GMG^ for symmetric systems as a function of the energy of the solid surface. It seems that, regardless of *σ*_g_, *N*_g_ and the ratio *N*_m_/*N*_g_, the strength of the polymer–solid interactions (wetting situation) has a minor effect on PMF^GMG^. The PMFs become less pronounced with enhanced wetting situation, albeit the effect is minor.

### 4.4. Interacting Grafted Surfaces in Vacuum (GVG)

**Figure 13:**
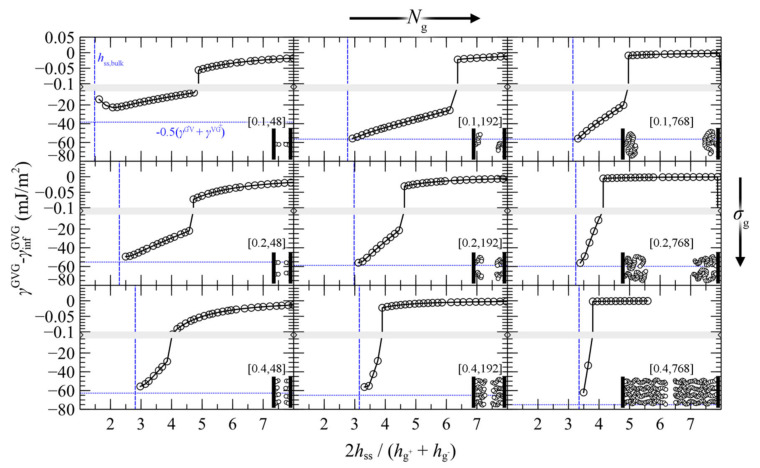
Potential of mean force for approaching grafted surfaces in vacuum (GVG) with the SL-SGT EoS. Values of *σ*_g_ (nm^−2^) and *N*_g_ are indicated in brackets. Horizontal blue lines mark minus the average surface free energy of the individual grafted films. Vertical blue lines mark the thickness *h*_ss_, that would correspond to the total mass of grafted polymer at bulk density. Insets: each bead denotes a segment of 24 PS monomers. Bands denote scale changes along the axes.

**Figure 14:**
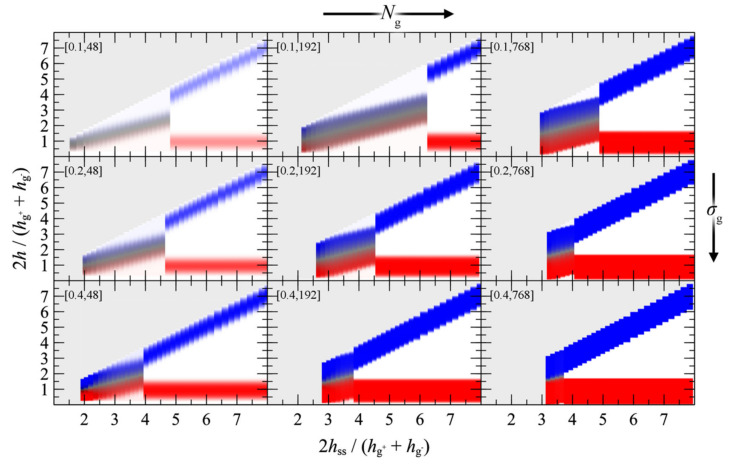
Reduced density distributions corresponding to the PMF^GVG^ panels in Figure 13, as functions of the plate-plate distance (abscissa) and the distance from the left wall (ordinate) in reduced units. Red/blue colors correspond to regions with high density (*φ_c_* = 1) of *c* = g^–^, g^+^ chains. White corresponds to vacuum. Grey denotes regions which lie outside the modeled domain or have not been evaluated at all. Labels in brackets denote *σ*_g_ (nm^−2^) and *N*_g_.

The present section discusses evaluations of the PMF^GVG^ of approaching grafted surfaces separated by vacuum (GVG). Figure 13 presents evaluations of PMF^GVG^ with *N*_g_ ranging from 24 to 768 and *σ*_g_ from 0.1 to 0.4 nm^–2^, whereas Figure 14 illustrates the corresponding density distributions for these cases. LW conditions have been used for surface-segment interactions.

The evolution of PMF^GMG^ with decreasing plate-plate distance can be classified in three distinct regimes:

1. For large separation distances the adjacent polymer brushes interact weakly with each other and the dominant contribution to PMF^GVG^ arises due to polymer/solid and solid/solid Hamaker interactions (e.g., compare with the dotted lines in Figure 6b–d).
2. Below a critical plate-plate distance, PMF^GVG^ decreases abruptly, indicating the manifestation of a phase transition where the adjacent brushes interpenetrate each other and form a single film in the central region of the system. In addition, low density regions are formed in the vicinity of the solid surface, indicating that the brushes have been stretched significantly towards the bulk region.
3. Decreasing the plate-plate separation further makes the brushes more compact. The low-density regions next to the solid become suppressed, until the free energy becomes commensurate to minus the mean surface free energy of the isolated brushes, $-0.5\left(\gamma^{G}{}^{{}^{-}V}+\gamma^{{\mathrm{VG}}^{+}}\right)$. Further squeezing of the brushes leads to increased reduced densities above unity, as indicated by the vertical dashed lines in Figure 13.

**Figure 15:**
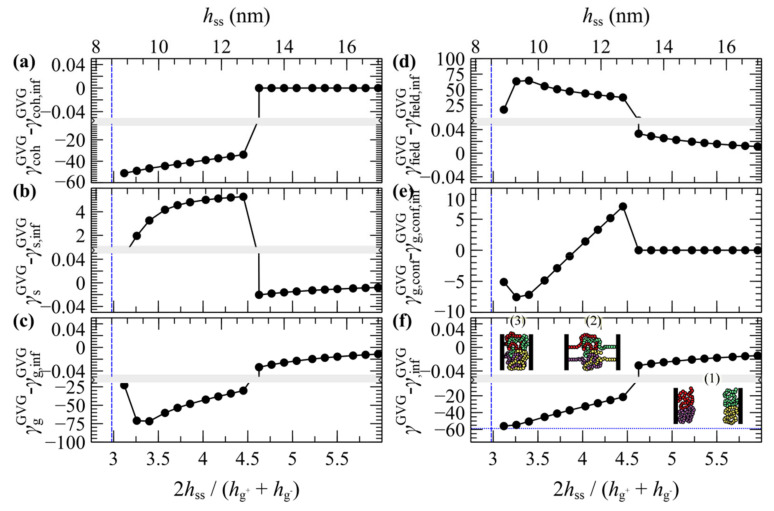
Free energy partial contributions to the potential of mean force for two approaching grafted surfaces with *σ*_g_ = 0.2 nm^–2^ and *N*_g_ = 192 in vacuum: (**a**) cohesive interactions, (**b**) polymer-solid interactions, (**c**) entropic contribution from grafted chains, (**d**) density-field interactions, (**e**) stretching contribution from grafted chains and (**f**) total grand potential. The vertical lines denote plate-plate distances where the reduced density exceeds unity. The horizontal dotted line in (**f**) depicts $-0.5\left(\gamma^{G^{-}V}+\gamma^{{\mathrm{VG}}^{+}}\right)$. The insets in (**f**) depict configurations across the 1st, 2nd and 3rd regime. Bands denote scale changes along the axes.

The chain configurations across these regimes are illustrated schematically in the inset of Figure 15f.

A more detailed picture can be unveiled by inspecting the evolution of individual contributions to the energy terms shown in Figure 15. The thermodynamics of the merger seems to be dominated by cohesive interactions. According to Figure 15a, below some critical distance the abrupt drop of the cohesive term ($\gamma_{\mathrm{coh}}^{\mathrm{GVG}}$) indicates the enthalpic gain upon film merging. At the same time, the more positive polymer–solid interactions in Figure 15b ($\gamma_{s}^{\mathrm{GVG}}$) indicate the enthalpic penalty due to the departure of a large portion of the brushes from the solid surface. The term associated with the entropy of the grafted chains ($\gamma_{g}^{\mathrm{GVG}}$) in Figure 15c is of particular interest. At first glance, it does not reflect the entropic penalty due the stretching of the grafted chains. However, this is attributable to the fact that $\gamma_{g}^{\mathrm{GVG}}$ does not reflect the total conformational contribution to the grand potential, since it is evaluated in the presence of the field [46]. The conformational component of the grafted chains can be retrieved as follows [46]:

(36) $$\gamma_{g,\mathrm{conf}}^{\mathrm{GVG}}=\gamma_{g}^{\mathrm{GVG}}+\gamma_{g,\mathrm{field}}^{\mathrm{GVG}}$$

with $\gamma_{g,\mathrm{field}}^{\mathrm{GVG}}$ being the field experienced by the grafted chains:

(37) $$\gamma_{g,\mathrm{field}}^{\mathrm{GVG}}S_{\mathrm{solid}}=-\int_{R}dr\left\{\left(\rho_{g^{-}}(r)+\rho_{g^{+}}(r)\right){w^{\prime }}_{\mathrm{ifc}}(r)\right\}$$

Indeed, as indicated in Figure 15e, the conformational free energy of the grafted chains increases abruptly below a critical distance (entropic penalty due to stretching) and then decreases with decreasing ${\tilde{h}}_{\mathrm{ss}}$, as the film becomes more compact and the grafted chains become less stretched.

In summary, the manifestation of the phase transition depends on an interplay among three dominant factors:

- An enthalpic gain due to the lower surface area of the merged brushes that increases with increasing surface tension.
- An enthalpic loss due to the detachment of the grafted film from the surface that depends on the polymer/solid interactions.
- A conformational penalty due to chain stretching.

As far as the equilibrium plate-to-plate distance after the merger is concerned, for low *σ*_g_*N*_g_ products this is on the order of 1.5 ${\tilde{h}}_{\mathrm{ss}}$, or $0.75(h_{g^{+}}+h_{g^{-}})$. In this case the plates come considerably closer than the sum of the individual root mean squared brush thicknesses, since the brushes lie in the mushroom regime and can interpenetrate each other easily. In the limit of large *σ*_g_*N*_g_, however, the brushes are more compact, and therefore one could make meaningful predictions using Alexander’s model for incompressible brushes (see Appendix A) [64,65]. Indeed, the denser brushes investigated here become compact (*ρ* > *ρ*_seg,bulk_) at separation distances on the order of ${\tilde{h}}_{\mathrm{ss},\min}\sim 2\sqrt{3}\sim 3.4$, e.g., compare with the vertical line in the bottom-right panel of Figure 13.

Overall, the effect of asymmetry on the equilibrium distance is expected to be minor. Indeed, according to Figure 13, brushes with equal *N*_g_*σ*_g_ products become compact at similar distances. For example, compare the case (*σ*_g_,*N*_g_) = (0.4 nm^–2^, 48) with (0.1 nm^–2^, 192) and the case (0.4 nm^–2^, 192) with (0.1 nm^–2^, 768).

## 5. Discussion

All of the above results concerning the potential of mean force between two planar surfaces (or equivalently of particles with radius large in relation to the chain dimensions) imply some design rules that one can be guided by when addressing such nanostructured systems.

As mentioned before, addressing the system of bare surfaces can give us an insight on what happens in the limit of very low grafting density. At moderate distances the behavior of the PMF depends on an interplay between the strength of the solid–polymer, polymer–polymer, and solid–solid interactions, as quantified by the wetting/spreading phenomena taking place at a single solid/polymer interface. For low wetting conditions (*θ* > 90°), the PMF becomes weakly repulsive from distances of the order of 3.5–10 nm, but the polymer film is metastable with respect to the chains evacuating the gap and the solid surfaces snapping into direct contact with each other. The spontaneous manifestation of cavities can lead to eventual collapse of the surfaces, leading to agglomeration. This is a manifestation of the allophobic dewetting phenomenon observed in the low wetting situation examined here (see SL-SGT (LW) case). On the contrary, for high wetting (*θ* < 90°) and perfect wetting (spreading) conditions, the PMF is practically zero at large distances and starts rising steeply below ca. 2 nm. A free energy barrier on the order of 20–80 mJ/m^2^ has to be overcome for the solid surfaces to come into direct contact. The system is stabilized in relation to the solid surfaces sticking to each other, and becomes trapped in a potential well with a depth in the order of ca. 20 mJ/m^2^ with respect to a melt-free system, e.g., with respect to the dotted lines shown in Figure 6. Matrix chains adhering to the solid surfaces resist compression and screen the solid–solid attractive interactions, as if they were grafted. The potential of mean force between bare solid surfaces does not depend strongly on the length of matrix chains; therefore, varying the molecular weight of matrix chains does not have a significant effect on the stability of the system.

In the case of low wetting of the solid by the polymer, grafted chains are necessary to stabilize the dispersion. In particular, when the grafted chain lengths and grafting densities are sufficiently high (e.g., as shown in Figure 12), the grafted chains effectively screen the solid–solid interactions, preventing the two plates from approaching each other at a distance where they would experience the full depth of the plate-plate potential. In many cases, PMF can become strictly repulsive, but a prerequisite for this is that the grafted chains be longer than the matrix chains. The longer the grafted chains in comparison to the matrix chains, the steeper the repulsive potential of mean force that develops and the longer the range over which it manifests itself. Short matrix chains are able to penetrate the brush of long grafted chains and swell it, increasing the range of the repulsive interaction. In this case, the most important design rule that has to be met, as already found experimentally [68,69,70,71,72], is that the grafted chains need to be larger the matrix chains, $N_{m}/N_{\bar{g}}\leq 1$. On the other hand, when matrix chains start becoming larger than the grafted ones, then immediately an attractive well is exhibited in the potential of mean force of the system (autophobic dewetting); solvent conditions become worse for the grafted chains. Furthermore, this behavior is intensified at high surface grafting densities. In these cases, the density of grafted chain segments near the interface is so high, that matrix chains are not able to penetrate into the region occupied by grafted chains, even if they have lower molecular weight than grafted chains. Thus, the use of excessive grafting densities is to be avoided for the purposes of steric stabilization, even if *N*_g_ > *N*_m_.

For the asymmetric cases, where the grafting density or the grafted chain molecular weight of grafted chains differs on each silica plate, it seems that the introduction of asymmetries does not give rise to a minimum in the potential of mean force, as long as deviations from the symmetric system are small, and the effective ratio $N_{m}/N_{\bar{g}}$ from Equation (33) is fixed. Individually, adjusting the asymmetry on the grafting density or on the molecular weight of grafted chains does not alter significantly the potential of mean force, with the former having a stronger influence. This implies that, when experimentalists are trying to stabilize dispersion of the two slabs (or large grafted particles) in a melt, there is some room for deviation from symmetry, especially as regards the molecular weight of grafted chains. On the other hand, when there are larger discrepancies between both the grafting densities and the molecular weight ratios, then the system will eventually exhibit an attractive well. Again, this phenomenon is more pronounced when the molecular weight of matrix chains increases.

**Figure 16:**
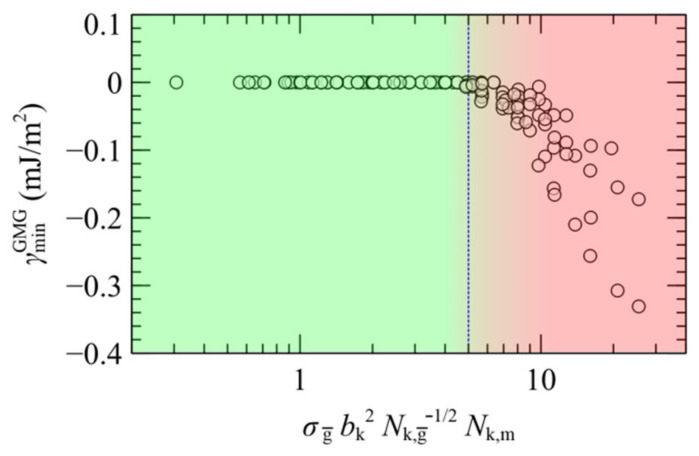
Well depth of PMF^GMG^ as a function of $\sigma_{\bar{g}}b_{k}{}^{2}N_{k,\;g\;¯}{}^{-1/2}N_{k,m}$. Green/red shades illustrate regimes with repulsive/attractive interactions between the opposing plates. The vertical dashed line is a guide to the eye.

The characteristics of the well depth of the PMF^GMG^ reflect the “softness” of the brushes as well as the associated tendency to penetrate into each other. [22] Wrapping together all the parameters which influence the attractive well of the PMF^GMG^ between the two plates, one can generate empirical design rules regarding the prediction of stable configurations of opposing plates (membranes or fine particles) as a function of the mean grafting density, the chain length of grafted chains and the chain length of matrix chains. According to Hasegawa et al. [25], PMF is expected to become repulsive for $\sigma_{g}\leq b_{k}{}^{-2}N_{k,\;\bar{g}}{}^{-1/2}$, with $N_{k,c}=N_{c}C_{\infty }l_{c-c}{}^{2}/b_{k}{}^{2}$ being the number of Kuhn segments that comprise a type *c* chain. In addition, as it has been demonstrated in Section 4.3, PMF^GMG^ becomes more attractive with increasing chain length of the matrix chains with respect to the effective length of grafted chains, $N_{m}/N_{\bar{g}}$ (or $N_{k,m}/N_{k,\;\bar{g}}$ in Kuhn units). Note that the effective ratio, $N_{m}/N_{\bar{g}}$, takes account of the asymmetry in both $\sigma_{g^{\pm }}$ and $N_{g^{\pm }}$. Putting all these together, it would be instructive to present the depth of the attractive well as a function of the dimensionless quantity, $\sigma_{\bar{g}}b_{k}{}^{2}N_{k,\;\bar{g}}{}^{1/2}\left[N_{k,m}/N_{k,\;g\;¯}\right]$, or $\sigma_{\bar{g}}b_{k}{}^{2}N_{k,\;\bar{g}}{}^{-1/2}N_{k,m}$, for simplicity. Such comparisons are shown in the master plot of Figure 16 against all the data gathered here for both symmetric and the asymmetric surfaces.

According to Figure 16 the attractive interactions become negligible for $\sigma_{\bar{g}}b_{k}{}^{2}N_{k,\;\bar{g}}{}^{-1/2}N_{k,m}$< 5, therefore for such combinations the surfaces are expected to stabilize. For larger values, on the other hand, in most cases the plates stick to each other (aggregation). Nevertheless, we must take into account that in the limit of very small average grafting density, the system will be led towards the case of bare solid plates in contact with vacuum, thus the melt is expected to evacuate the gap and the plates will spontaneously come in contact to each other. According to our calculations, the PS melt remains stable even for low grafting densities on the order of 0.1 nm^−2^ ($\sim 0.33\;b_{k}{}^{-2}$), thus for these systems the region of stability could be traced along the range $0.33\leq \sigma_{\bar{g}}b_{k}{}^{2}\leq 5\;N_{k,\;\bar{g}}{}^{1/2}N_{k,m}{}^{-1}$. Our findings conform with experimental studies concerning systems of the same [69,70,71] or similar [68,70,72] chemical constitution and with theoretical works [22,25,67], whilst accounting for the effect of asymmetry as well.

## 6. Conclusions

The present work investigates the thermodynamics of single and opposing silica surfaces, either bare or grafted, under various wetting conditions and solvent situations. The calculations have been realized with the self-consistent field theory. For comparison purposes, the nonbonded dispersive interactions are based on the Helfand (HFD), or the Sanchez-Lacombe (SL) free energy density. The polymer–solid and solid–solid interactions were described with the Hamaker potential in conjunction with a ramp potential. By adjusting the depth of the ramp potential, we examined cases with low wetting (*θ* = 140–160°, depending on the EoS), high wetting (*θ*~67°), and perfect wetting (spreading), whereas for each case we derived the corresponding macroscopic wetting functions.

We investigated situations of grafted surfaces in contact with vacuum or a melt phase, in terms of their structure and interfacial free energies. In vacuum conditions, the overall shape of the profiles and the brush thickness are dictated by the product, *σ*_g_*N*_g_; the brushes appear collapsed and feature a sigmoid region of ca. 1 nm at the vacuum interface. In melt conditions, the brushes expand significantly towards the bulk phase. Nonetheless, in the high *σ*_g_*Ν*_g_ regime, the scaling of the brush thickness conforms to the prediction of Alexander’s model for incompressible brushes, independent of whether they are in contact with vacuum or melt. The EoS and the wetting conditions appear to have a minor effect on the overall structure of the single brushes.

We performed a systematic and comprehensive investigation of PMF over a broad parameter space in terms of the following aspects:

- Grafting densities; very low (*σ*_g_ = 0, bare surfaces), moderate and high.
- Asymmetry regarding the grafting densities and grafted chain lengths.
- Solvent conditions; good ($N_{m}/N_{\;g\;¯}<1$), theta ($N_{m}/N_{\bar{g}}=1$), bad ($N_{m}/N_{\bar{g}}<1$ ), and very bad (vacuum) solvents.
- Wetting degree; low, high and perfectly wetted interfaces.

In doing so, we isolated the key parameters that are important to the resulting PMF and the tendencies of the opposing surfaces (or the corresponding fine particles) to agglomerate.

Initially, we evaluated the potential of mean force (PMF) between two bare silica plates with melt between them, each time varying the wetting situation. HFD was found inadequate to describe these systems because it cannot describe the occurring cavitation, whereas SL can. The LW films collapse for surface-surface distances lower than ca. 3.5 nm. In contrast, the HW and PW films are retained during the whole compression experiment. A difference between HW and PW is that the latter are stable with respect to cavitation down to thicknesses of ca. 1.5 nm, since the minimum due to Hamaker potential at low distances is located higher than the plateau free energy at large distances. In these situations, the matrix chains adhere strongly to the surfaces—as if they were grafted—and are able to effectively screen the solid–solid attractive interactions.

Regarding the PMF between grafted surfaces with melt in between them, a general conclusion is that with decreasing molecular weight of matrix chains, the solvent conditions for the grafted chains are enhanced, meaning that the grafted chains prefer to interact with the chains of the melt than with themselves. This behavior is manifested through the repulsive potential of mean force between the two grafted solid plates. On the other hand, when the melt chains are of much larger molecular weight than the grafted chains or when vacuum exists between the two grafted plates, then the grafted chains prefer to interact with themselves, adopting the corresponding configurations. The effect of asymmetry is minor when adjusting the lengths and grafted chains individually, and given that the effective ratio $N_{m}/N_{\bar{g}}$ is fixed, indicating that there is some room for error during the experimental processes. The role of wetting and the employed EoS appear to have a minor effect on PMF. Based on our calculations we find a region of stability (steric stabilization) traced along the following range:$0.33\leq \sigma_{\bar{g}}b_{k}{}^{2}\leq 5\;N_{k,\;\bar{g}}{}^{1/2}N_{k,m}{}^{-1}$. This predictive behavior is in agreement with relevant studies reported in literature [22,25,67,68,69,70,71,72].

Regarding the grafted silica plates without melt chains in the space between them, it would be interesting to confirm experimentally the predictions derived here on how their equilibrium distance is affected by the grafting density and the matrix to grafted chains molecular weight ratio. For the lowest grafting densities investigated here the equilibrium distances are on the order of 1.5 the mean brush thickness, whereas with increasing *N*_g_ and *σ*_g_ the brushes become more compact and the equilibrium distance increases to about $2\sqrt{3}$ mean brush thickness.

Future prospects of the study include the investigation of the potential of mean force between opposing surfaces (or even pairs of nanoparticles) embedded in melt or vacuum phases (particle solids) using the three-dimensional version of *RuSseL* [52]. These calculations will allow us to investigate situations with homogeneous, and inhomogeneous distributions of grafted points across the surface of the NP (plane).

## Figures and Tables

**Table 1:** Parameters of the self-consistent field theory (SCFT) calculations.

	Parameter	Value	Reference
System	*T*	500 K	
	$r_{\mathrm{ref},q=0}^{}$	0.05 nm	-
	$r_{g,i_{g},q=0}^{}$	0.05 nm	-
Chain stiffness	*b*_k_	1.83 nm	[15]
	*l*_C-C_	0.154 nm	-
	γ	0.829	[53]
	*m*_monomer_	52.08 g/mol	-
Hamaker	*h*_HS_	~0.4 nm	-
	$\sigma_{\mathrm{PS}}$	0.37 nm	[15]
	$\sigma_{{\mathrm{SiO}}_{2}}$	0.30 nm	[15]
	$A_{\mathrm{PS}}$	5.84 × 10^−20^ J	[15]
	$A_{{\mathrm{SiO}}_{2}}$	6.43 × 10^−20^ J	[15]
Ramp potential	*v*_ramp_ (LW)	0.0	
	*v*_ramp_ (HW)	–2.481 × 10^−20^ J	Fitted to *W*_A_ = 38.8 mJ/m^2^ from [54]
	*v*_ramp_ (PW)	–3.975 × 10^−20^ J	Fitted to *W*_A_ = 71.1 mJ/m^2^ from [54]
	*σ*_ramp_	1.28 nm	
Helfand	$\kappa_{T=500K}$	3.97 (GPa)^−1^	[55,56]
	$\rho_{\mathrm{mass},\mathrm{bulk}}$	953 kg/m^3^	[15]
Sanchez Lacombe	ρ*	1105 kg/m^3^	[57]
	P*	357 MPa	[57]
	T*	735 K	[57]
Square Gradient	*κ* $\tilde{\kappa }$	0.2233·10^–66^ J m^5^ 0.55	[44]
Edwards Diffusion	Δh	0.05 nm	[46]
	ΔN	0.25	[46]
	$\Delta {w^{\prime }}_{\mathrm{ifc}}^{\mathrm{tol}}$	10^−6^ $k_{B}T$	-

**Table 2:** Interfacial energies and wetting functions for *N*_m_ = 384 in units of mJ/m^2^.

EoS	Wetting	*γ* ^VM^	*γ* ^SM^	*σ*^SV^–*σ*^SM^	*W*_A_	*W*_S_	*W*_I_	*W*_C_	*θ* (°)
HFD	low (LW)	28.85	21.97	–21.97	6.88	–50.81	–21.97	57.70	139.6
SL-SGT	low (LW)	27.89	26.02	–26.02	1.86	–53.91	–26.02	55.77	158.9
SL-SGT	high (HW)	27.89	–10.91	10.91	38.8 [54]	–16.97	10.91	55.77	67.0
SL-SGT	Full (FW)	27.89	–43.21	43.21	71.1 [54]	15.33	43.21	55.77	-

## Data Availability

Data available on request.

## Supplementary Materials

The following are available online at https://www.mdpi.com/article/10.3390/polym13081197/s1, Section S1. Optimization of Helfand’s isothermal compressibilities, Table S1, Figure S1; Section S2. Special difficulty in converging longer chains in polymer/vacuum interphases, Figure S2, Figure S3; Section S3. Wetting functions as a function of chain length for SL-SGT(LW), Figure S4; Section S4. Brush thickness as a function of wetting, Figure S5; Section S5. Potential of mean force of opposing bare surfaces starting from zero field (w_ifc_ = 0), Figure S6; Section S6. Asymmetric surfaces in GMG systems for (*σ*_g_-, *N*_g_-) equal to (0.2 nm^–2^, 384) and (0.4 nm^–2^, 96), Figure S7, Figure S8.

## Appendix A

In planar surfaces, the edge of a brush with *N*_g_ and *σ*_g_ lies at [46],

(A1) $$h_{\mathrm{edge},g}^{}=\sigma_{g}N_{g}/\rho_{\mathrm{seg},\mathrm{bulk}}$$

thus, the separation distance of two Alexander brushes (with $\sigma_{g^{-}},N_{g^{-}}\;\mathrm{and}\;\sigma_{g^{+}},N_{g^{+}}$) in contact is:

(A2) $$h_{\mathrm{ss},\min}^{}=h_{{\mathrm{edge},g}^{-}}^{}+h_{{\mathrm{edge},g}^{+}}^{}=\frac{\sigma_{g^{-}}N_{g^{-}}+\sigma_{g^{+}}N_{g^{+}}}{\rho_{\mathrm{seg},\mathrm{bulk}}}$$

The root mean squared brush thickness of an Alexander brush equals ${\langle h_{g}{}^{2}\rangle }^{0.5}=h_{\mathrm{edge},g}^{}/\sqrt{3}$, [46]; hence, in terms of reduced units $h_{\mathrm{ss},\min}^{}$ becomes:

(A3) $${\tilde{h}}_{\mathrm{ss},\min}^{}=2\frac{h_{{\mathrm{edge},g}^{-}}^{}+h_{{\mathrm{edge},g}^{+}}^{}}{{\langle h_{g^{-}}{}^{2}\rangle }^{0.5}+{\langle h_{g^{+}}{}^{2}\rangle }^{0.5}}=2\frac{h_{{\mathrm{edge},g}^{-}}^{}+h_{{\mathrm{edge},g}^{+}}^{}}{\frac{h_{{\mathrm{edge},g}^{-}}^{}}{\sqrt{3}}+\frac{h_{{\mathrm{edge},g}^{+}}^{}}{\sqrt{3}}}=2\sqrt{3}$$

which is about 3.4.
