# Supplementary material for: Potential of Mean Force between Bare or Grafted Silica/Polystyrene Surfaces from Self-Consistent Field Theory
Source: Polymers (Basel). 2021 Apr 7;13(8):1197. doi: 10.3390/polym13081197 (PMC8068000; doi:10.3390/polym13081197)
Supplement: Supplementary file 1 [file polymers-13-01197-s001.pdf]

polymers

Article

Potential of Mean Force between Bare or Grafted Silica/Polystyrene Surfaces from Self-Consistent Field Theory

Supporting Information

Aristotelis P. Sgouros[1,*,†], Constantinos J. Revelas [1,†], Apostolos T. Lakkas[1] and Doros N. Theodorou[1,*]

[1] School of Chemical Engineering, National Technical University of Athens (NTUA), GR-15780 Athens, Greece; cjrevelas@gmail.com (C.R.)
* Correspondence: arissgouros@gmail.com, Tel.: +30 210 772 3216 (A.S.); doros@central.ntua.gr, Tel.: +30 210 772 3157 (D.T.)
† These authors contributed equally to this work.

S1. Optimization of Helfand's isothermal compressibilities

The SL EoS has been shown [1,2] to reproduce accurately the experimental surface tensions of several polymer melts when used in conjunction with SGT [3] (e.g., compare markers for SL and SL-SGT in Figure **S1h**). In addition, the local structure, as described by the segmental density profiles, (e.g., Figure **S1b**) conforms with predictions from atomistic simulations.[1,4]

Regarding HFD, its free energy density is dictated by two free parameters; namely the isothermal compressibility and the bulk density of the melt, as shown in eq (19). A simple way to determine these parameters is to either retrieve relevant experimental values or fit them in a way that reproduces the surface tension of the fluid. Nonetheless, setting these parameters to constant values leads to inaccurate qualitative predictions for the surface tension with varying chain molar mass or temperature. For example, in Figure **S1h,** the predicted surface tension from HFD (with $\kappa_{T,\mathrm{exp}}$=3.97 GPa^{-1} and $\rho_{\mathrm{mass,bulk}}$ = 953 g/cm^3) appears to be a decreasing function of chain length, whereas the opposite trend has been observed from simulations[5] and experiments[2,6]; i.e., the surface tension is an increasing function of N_m. This is reasonable, since cohesive interactions in the polymer melt are enhanced with increasing chain length.

A reasonable approach for setting these parameters would be to set them equal to the ones predicted by the SL model for a specific chain length and temperature, using the following eq (S1).[7]

$$\kappa_{T,\mathrm{SL}}{}^{-1} = \tilde{T}P^* \tilde{\rho}_{\mathrm{seg,bulk}}{}^2 \left(\frac{1}{1-\tilde{\rho}_{\mathrm{seg,bulk}}} + \frac{1}{\tilde{\rho}_{\mathrm{seg,bulk}} r_{\mathrm{SL}} N_\mathrm{m}} - \frac{2}{\tilde{T}} \right) \quad \text{(S1)}$$

Using HFD with $\kappa_{T,\mathrm{SL}}$ from eq (S1) (see **Table S1**), even though producing good qualitative behavior with varying N_m and T, leads to rather high values of the surface tension (see squares in Figure **S1h**); the deviation becomes larger with the addition of SGT (see purple crosses in Figure **S1h**).

An alternative way to derive these parameters would be to fit them directly into experimental or theoretically predicted surface tensions (from a suitable EoS such as SL-SGT) for each chain length. **Table S1** illustrates the optimized isothermal compressibilities (via the Secant method) for HFD and HFD/SGT for each chain length, according to the values of surface tension obtained via the SL-SGT model (i.e., compare SL-SGT with HFD/$\kappa_{T,\mathrm{opt}}$ and HFD/SGT, κ_{T_opt} in Fig. Figure **S1h**). The compressibilities were optimized using the Secant method.

It is mentioned that, even though the reduced density profiles do not appear to be dependent on the chain length, the actual density profiles do change, as indicated by the chain length-dependent $\rho_{\mathrm{seg,bulk}}$ values in **Table S1**; it is the shape of these profiles that remains the same.

Table S1. Bulk densities and compressibilities from the SL EoS, and optimized compressibilities for the HFD, HFD/SGT EoS.

N_m	$\rho_{mass,bulk}$ (g/cm³)	$\kappa_{T,SL}$ (GPa⁻¹)	$\kappa_{T,HFD,opt}$ (GPa⁻¹)	$\kappa_{T,HFD/SGT,opt}$ (GPa⁻¹)
24	0.93997	1.50804	4.56055	15.96570
48	0.94216	1.46935	4.36072	14.88736
96	0.94324	1.45060	4.26308	14.39257
192	0.94378	1.44137	4.21460	14.15635
384	0.94405	1.43679	4.18998	14.03992
768	0.94418	1.43451	4.17748	13.98211
1536	0.94425	1.43337	4.17119	13.95340

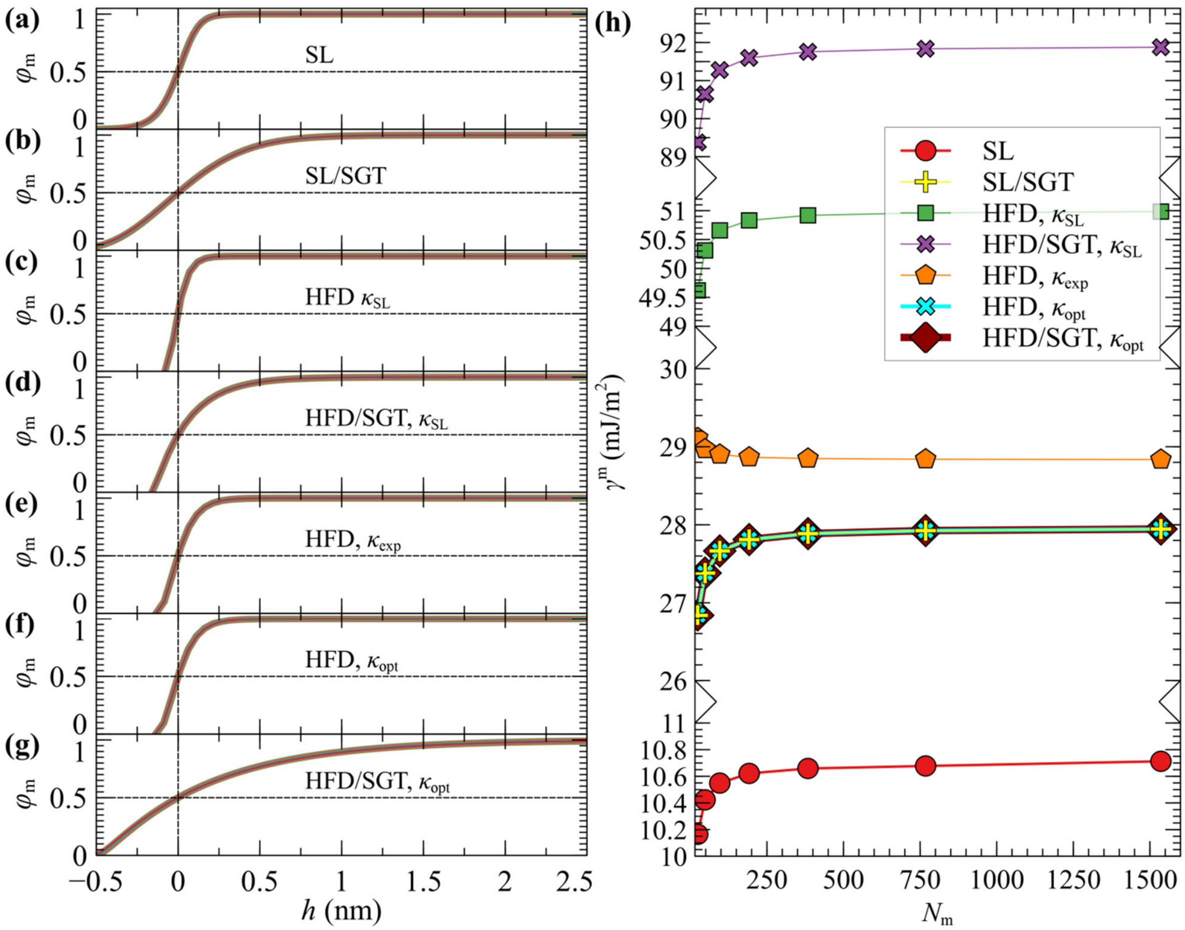

Figure **S1. (a-g)** Reduced density profiles at the free surface of a melt from SL and HFD (for various sets of isothermal compressibilities from Table **S1**. with and without SGT, for N_m = 24 up to 1536. The solid lines become thicker with increasing N_m, while the dashed lines are guides to the eye. **(h)** The surface tension from the corresponding profiles in panels (a-g).

S2. Special difficulty in converging longer chains in polymer/vacuum interphases

Figure **S2** depicts the reduced density profiles of an SM system after the first iteration of SCF, where $\mathbf{w}'_{\text{ifc,init}} = \mathbf{0}$ everywhere across the domain. Since the Edwards diffusion equation is solved under zero field, the same results are derived regardless of the EoS. The matrix chains are pushed further towards the bulk region with increasing N_m, and this makes the convergence more difficult, since the difference of the density profile from the converged one increases.

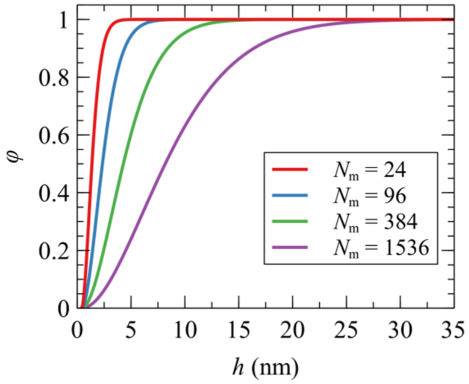

Figure S2. Reduced density profiles after the first iteration of the SCFT calculation, where $w_{\text{inf}} = 0$, everywhere across the domain.

Figure **S3a** (red line) depicts the evolution of the maximum error in the field, $w'_{\text{ifc,err}}$ for $N_m = 1536$ starting from zero field, $\mathbf{w}'_{\text{ifc,init}} = \mathbf{0}$. The error in the field decreases during the first ~10^5 steps and subsequently stabilizes at $w'_{\text{ifc,err}} \sim 10^{-3}\, k_B T$ for a large number of steps/iterations. The system is trapped in a metastable state as indicated by the apparent plateau of the free energy at ~27.8 mJ/m^2. The field increases slowly over the span of several millions of steps, and eventually converges ($w'_{\text{ifc,err}} \leq 10^{-7} k_B T$) to the correct solution at ~$2 \cdot 10^7$ steps. On the other hand, if we perform the same calculation, starting instead from a converged field for a lower chain length, we observe that the field converges ($w'_{\text{ifc,err}} \leq 10^{-7} k_B T$) after 10^5 iterations, and the free energy converges readily to its stable solution; see green lines in Figure **S3a and b**. The first calculation is ~100 times slower than the second one.

The same effect can be reproduced for small chain lengths as well. Figure **S3c and d**, depicts the evolution of $w'_{\text{ifc,err}}$ and γ^{SM} for systems that start from a "bad" field configuration (green lines; the field considering the metastable solution of $N_m = 1536$) and from $w'_{\text{inf}} = 0$ (red lines). The calculation requires up to 30 times more steps to converge when we start from the "bad" field configuration.

Based on the above tests, it seems that the best practice for evaluating melts with large chains is to perform continuation based on the converged fields from smaller chain lengths.

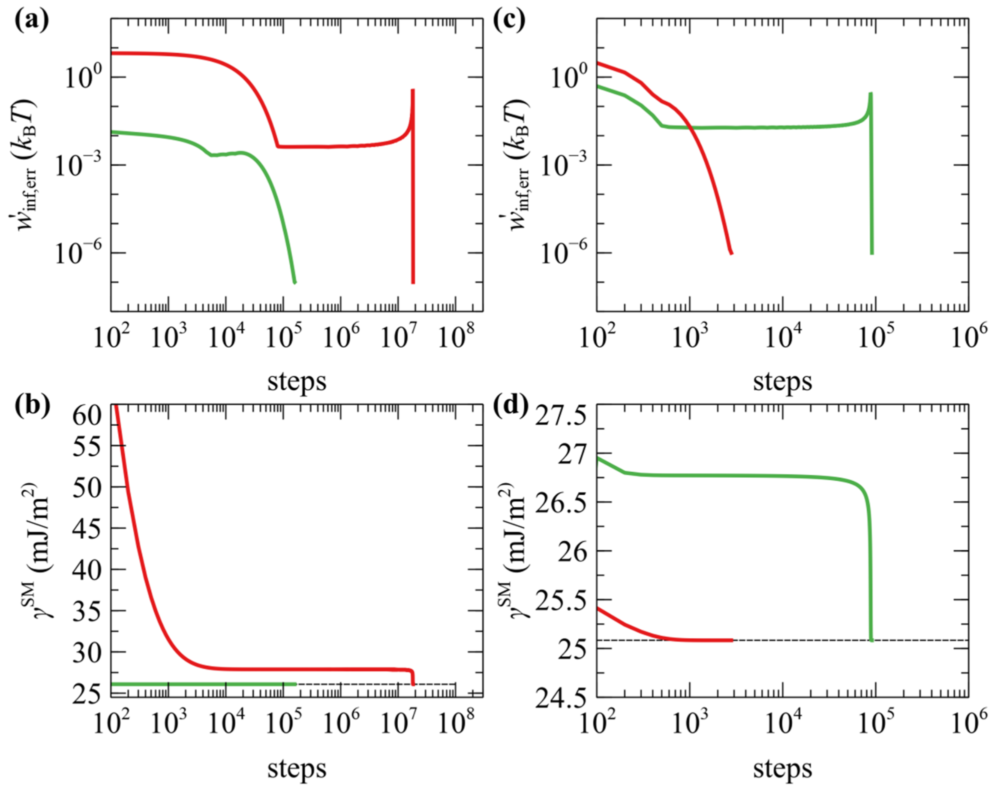

Figure S3. Evolution of the **(a)** maximum field error and **(b)** free energy of N_m = 1536 chains with a mixing fraction $w'_{inf,mix}$ = 0.000091, starting from zero field (red line) and from the converged field for N_m = 768 (green line). Evolution of the **(c)** field error and **(d)** free energy of N_m = 24 chains with a mixing fraction $w'_{inf,mix}$ = 0.000708, starting from zero field (red line) and from the field characteristic of the metastable configuration for N_m = 1536 (green line).

S3. Wetting functions as a function of chain length for SL-SGT(LW)

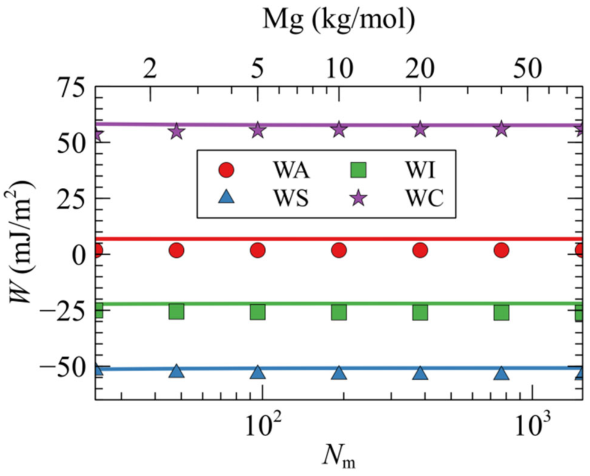

Figure S4. Wetting functions of the PS-Silica interphases with the SL-SGT (markers) and HFD (lines) in the absence of the ramp potential (low wetting). The wetting functions are the following: work of adhesion (red/circles), work of spreading (blue/triangles), work of immersion (green/squares), and work of cohesion (violet/stars).

S4. Brush thickness as a function of wetting

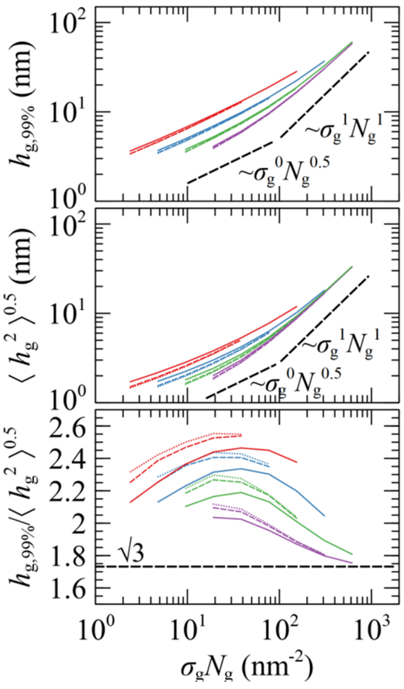

Figure S5. **(a)** $h_{g,99\%}$, **(b)** $\left\langle h_g^{\ 2} \right\rangle^{0.5}$, and **(c)** their ratio $h_{g,99\%} \big/ \left\langle h_g^{\ 2} \right\rangle^{0.5}$ compared to the scaling law $\sigma_g^1 N_g^1$ in GM systems with $N_g = N_m$. Different line styles denote interfaces with low (solid lines), high (dashed lines) and perfect (dots) wetting.

S5. Potential of mean force of opposing bare surfaces starting from zero field ($\mathbf{w}_{\mathrm{ifc}} = 0$)

Figure **S6** illustrates evaluations of PMF^{SMS} with HFD (a), and with SL-SGT under low (**b**, LW), high (**c**, HW) and perfect (**d**, PW) wetting situations. In these evaluations the field was initialized to zero across the domain, $\mathbf{w}'_{\mathrm{ifc,init}} = \mathbf{0}$. It appears the regardless of the compression method evaluations with HFD converge to the same solution; i.e., compare with Fig. **6a**. The evaluations with SL-SGT, on the other hand, are very sensitive to the compression method. In the case for LW films, below a critical plate-plate distance ($h_{\mathrm{ss}}^{\mathrm{crit}}$), PMF^{SMS} decreases abruptly, indicating a phase transition. $h_{\mathrm{ss}}^{\mathrm{crit}}$ scales with the radius of gyration as $h_{\mathrm{ss}}^{\mathrm{crit}} \sim 3\ R_{\mathrm{g}}$. During the first SCF iteration, the matrix chains are pushed further towards the central region of the domain with increasing N_{m} and form broad lower-density regions near the Dirichlet face, as shown in **Figure S2**. With decreasing plate-plate distance these low density regions overlap and promote cavitation.

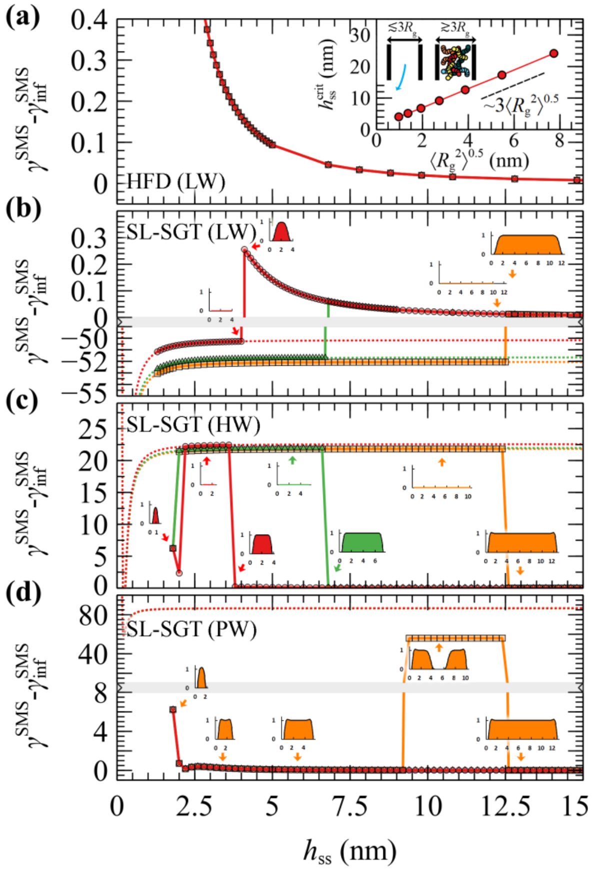

Figure S6. Potential of mean force, in mJ/m^2, for the system of approaching bare silica surfaces (SMS) obtained by the **(a)** HFD, **(b)** SL-SGT (LW), **(c)** SL-SGT (HW) and **(d)** SL-SGT (PW), for N_{m} = 24 (red), 96 (green), and 384 (orange). The initial configuration of the field was set to $\mathbf{w}'_{\mathrm{ifc,init}} = \mathbf{0}$. The inset in panel (a) depicts the critical plate-plate distance for matrix to evacuate the system against their radius of gyration when the first approach is used and the schematic therein depicts the film situation for h_{ss} larger and lower than ~3R_{g}. The inset graphs in (b-d) depict snapshots of the density profiles at plate-plate distances denoted by the arrows and their fill corresponds to the color of the corresponding chain length. Bands denote scale changes along the axes. The dashed lines display the Hamaker potential contribution to the solid-solid interaction, shifted by twice the solid/polymer adhesion tension.

Regarding the HW film in **Figure S6c**, it converges to a cavitated state at slightly lower h_{ss}^{crit} as compared to the LW one; for all distances higher than ca. 0.5 nm, this cavitated state is metastable relative to the polymer-filled state, unlike what happens in the LW case. Calculations on the PW films with $\mathbf{w}'_{ifc,init} = \mathbf{0}$, on the other hand, maintain the stable PW film in contact with the surfaces over the full range of h_{ss} examined in almost all cases (see **Figure S6d**). Only for $N_m = 384$ does the PW film in **Figure S6d** form a central cavity for ~9.2 nm < h_{ss} < 12.6 nm, converging to a less stable partially cavitated solution.

S6. Asymmetric surfaces in GMG systems for (σ_{g-}, N_{g-}) equal to (0.2 nm^{-2},384) and (0.4 nm^{-2},96).

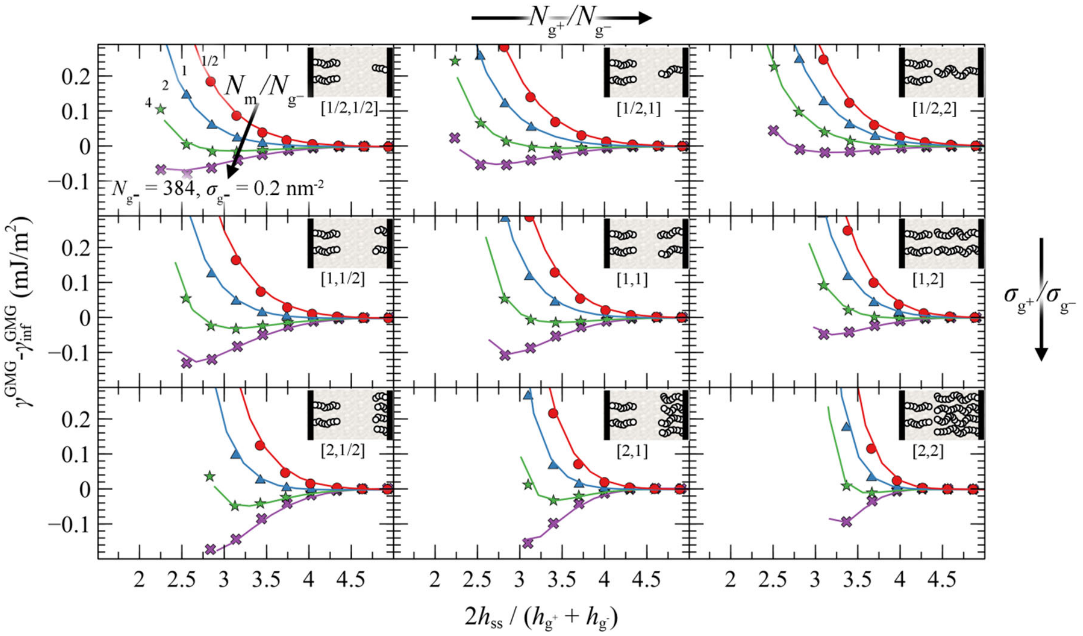

Figure S7. Potential of mean force against the reduced surface-surface distance for approaching grafted silica surfaces in a melt for $\sigma_{g^-} = 0.2$ nm^{-2} and $N_{g^-} = 384$. Colors correspond to evaluations for $N_m / N_{g^-} = 1/2$ (red), 1 (blue), 2 (green) and 4 (purple), whereas the legends denote the ratios $\sigma_{g^+} / \sigma_{g^-}$ and N_{g^+} / N_{g^-}, respectively. Lines and markers correspond to evaluations with the HFD and SL EoS, respectively. Inset: each bead denotes a segment of 96 PS monomers.

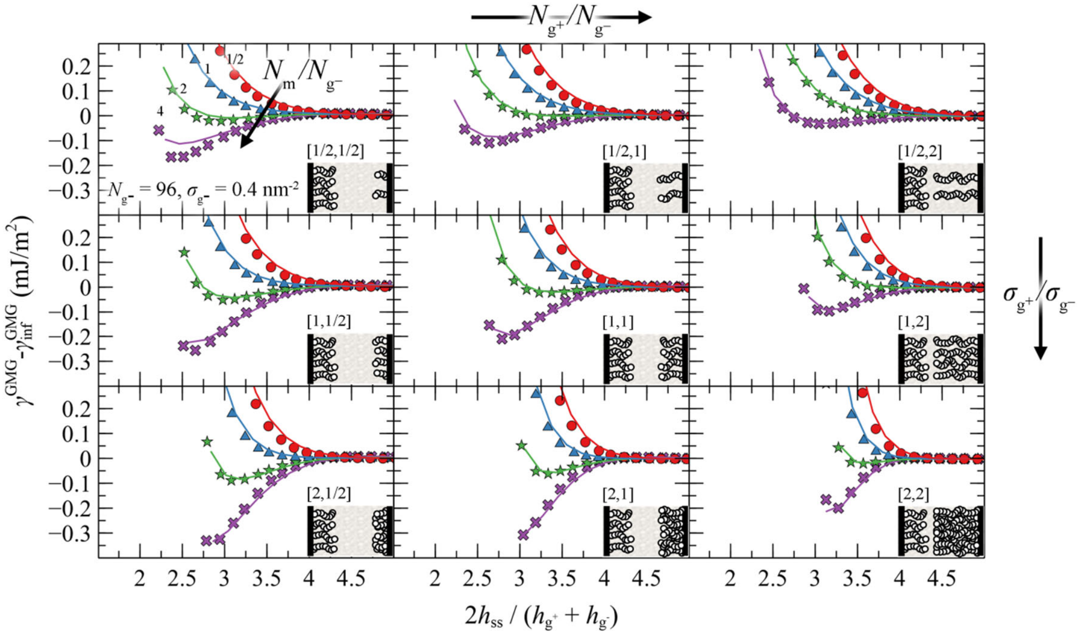

Figure S8. Potential of mean force against the reduced surface-surface distance for approaching grafted silica surfaces in a melt for $\sigma_{g^-} = 0.4$ nm^{-2} and $N_{g^-} = 96$. Colors correspond to evaluations for $N_m / N_{g^-} = 0.5$ (red), 1 (blue), 2 (green) and 4 (purple), whereas the legends denote the ratios $\sigma_{g^+} / \sigma_{g^-}$ and N_{g^+} / N_{g^-}, respectively. Lines and markers correspond to evaluations with the HFD and SL EoS, respectively. Inset: each bead denotes a segment of 24 PS monomers.

References

1. Lakkas, A.T.; Sgouros, A.P.; Theodorou, D.N. Self-Consistent Field Theory Coupled with Square Gradient Theory of Free Surfaces of Molten Polymers and Compared to Atomistic Simulations and Experiment. *Macromolecules* **2019**, *52*, 5337–5356, doi:10.1021/acs.macromol.9b00795.

2. Poser, C.I.; Sanchez, I.C. Surface tension theory of pure liquids and polymer melts. *J. Colloid Interface Sci.* **1979**, *69*, 539–548, doi:10.1016/0021-9797(79)90142-5.

3. Lin, H.; Duan, Y.Y.; Min, Q. Gradient theory modeling of surface tension for pure fluids and binary mixtures. *Fluid Phase Equilib.* **2007**, *254*, 75–90, doi:10.1016/j.fluid.2007.02.013.

4. Sgouros, A.P.; Lakkas, A.T.; Megariotis, G.; Theodorou, D.N. Mesoscopic Simulations of Free Surfaces of Molten Polyethylene: Brownian Dynamics/kinetic Monte Carlo Coupled with Square Gradient Theory and Compared to Atomistic Calculations and Experiment. *Macromolecules* **2018**, *51*, 9798–9815, doi:10.1021/acs.macromol.8b01873.

5. Sgouros, A.P.; Vogiatzis, G.G.; Kritikos, G.; Boziki, A.; Nikolakopoulou, A.; Liveris, D.; Theodorou, D.N. Molecular Simulations of Free and Graphite Capped Polyethylene Films: Estimation of the Interfacial Free Energies. *Macromolecules* **2017**, *50*, 8827–8844, doi:10.1021/acs.macromol.7b01808.

6. Rolo, L.I.; Caço, A.I.; Queimada, A.J.; Marrucho, I.M.; Coutinho, J.A.P. Surface tension of heptane, decane, hexadecane, eicosane, and some of their binary mixtures. *J. Chem. Eng. Data* **2002**, *47*, 1442–1445, doi:10.1021/je025536+.

7. Sanchez, I.C.; Lacombe, R.H. An elementary molecular theory of classical fluids. Pure fluids. *J. Phys. Chem.* **1976**, *80*, 2352–2362, doi:10.1021/j100562a008.
